# The Precise Structures and Stereochemistry of Trihydroxy-linoleates Esterified in Human and Porcine Epidermis and Their Significance in Skin Barrier Function: IMPLICATION OF AN EPOXIDE HYDROLASE IN THE TRANSFORMATIONS OF LINOLEATE*[Fn FN2]

**DOI:** 10.1074/jbc.M115.711267

**Published:** 2016-05-05

**Authors:** Takahito Chiba, Christopher P. Thomas, M. Wade Calcutt, William E. Boeglin, Valerie B. O'Donnell, Alan R. Brash

**Affiliations:** From the Departments of ‡ Pharmacology and; ¶ Biochemistry and the Vanderbilt Institute of Chemical Biology, Vanderbilt University School of Medicine, Nashville, Tennessee 37232 and; the § Systems Immunity Research Institute, School of Medicine, Cardiff University, Cardiff CF14 4XN, Wales, United Kingdom

**Keywords:** ceramide, epidermis, lipid oxidation, lipoxygenase pathway, mass spectrometry (MS), essential fatty acid, linoleic acid

## Abstract

Creation of an intact skin water barrier, a prerequisite for life on dry land, requires the lipoxygenase-catalyzed oxidation of the essential fatty acid linoleate, which is esterified to the ω-hydroxyl of an epidermis-specific ceramide. Oxidation of the linoleate moiety by lipoxygenases is proposed to facilitate enzymatic cleavage of the ester bond, releasing free ω-hydroxyceramide for covalent binding to protein, thus forming the corneocyte lipid envelope, a key component of the epidermal barrier. Herein, we report the transformations of esterified linoleate proceed beyond the initial steps of oxidation and epoxyalcohol synthesis catalyzed by the consecutive actions of 12*R*-LOX and epidermal LOX3. The major end product in human and porcine epidermis is a trihydroxy derivative, formed with a specificity that implicates participation of an epoxide hydrolase in converting epoxyalcohol to triol. Of the 16 possible triols arising from hydrolysis of 9,10-epoxy-13-hydroxy-octadecenoates, using LC-MS and chiral analyses, we identify and quantify specifically 9*R*,10*S*,13*R*-trihydroxy-11*E*-octadecenoate as the single major triol esterified in porcine epidermis and the same isomer with lesser amounts of its 10*R* diastereomer in human epidermis. The 9*R*,10*S*,13*R*-triol is formed by *S*_N_2 hydrolysis of the 9*R*,10*R*-epoxy-13*R*-hydroxy-octadecenoate product of the LOX enzymes, a reaction specificity characteristic of epoxide hydrolase. The high polarity of triol over the primary linoleate products enhances the concept that the oxidations disrupt corneocyte membrane lipids, promoting release of free ω-hydroxyceramide for covalent binding to protein and sealing of the waterproof barrier.

## Introduction

**FIGURE 1.**
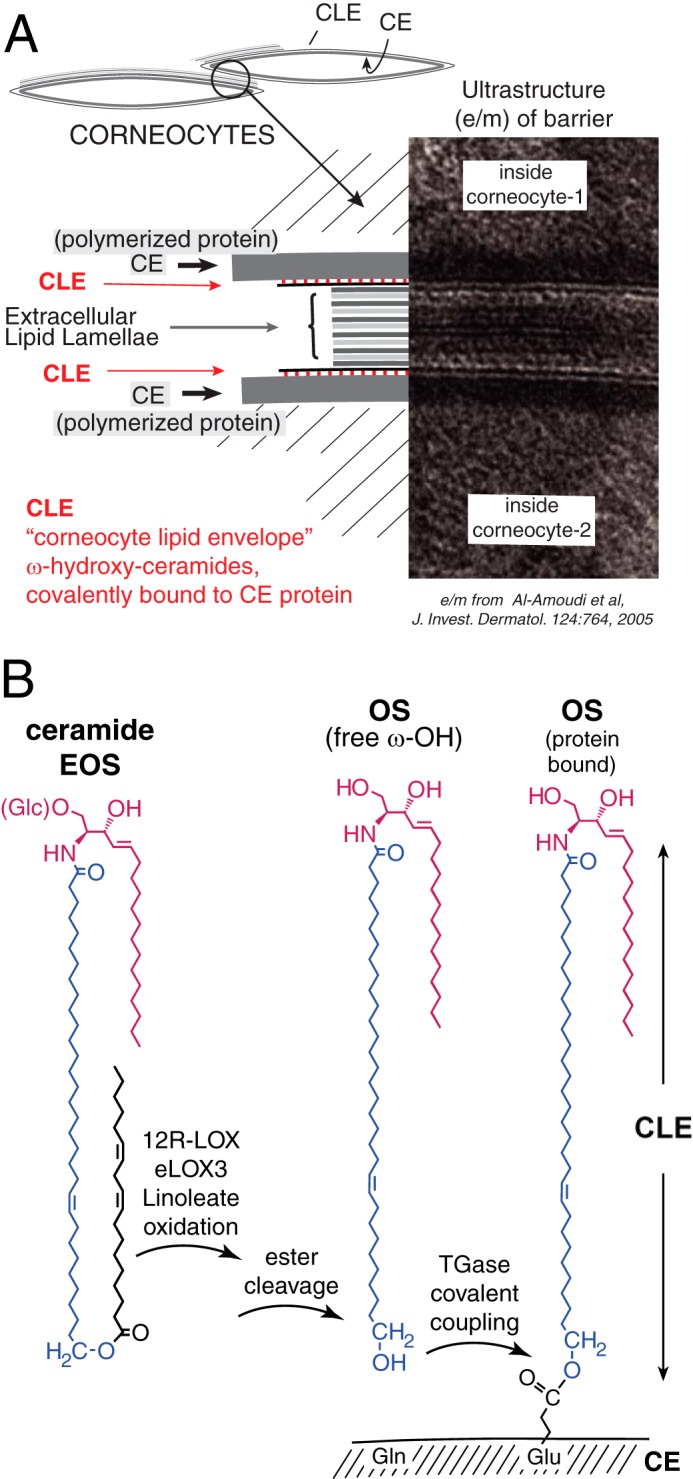
**Structures of the epidermal water barrier and formation of the CLE.** *A,* electron micrograph of the epidermal barrier with the parts illustrated in schematic style. In the barrier layer of the epidermis the corneocytes are melded together by fusion of three substructures: polymerized protein forming the corneocyte envelope (*CE*) on the periphery of each cell, extracellular lamellar lipids between cells, and the monolayer of covalently bound ceramides and fatty acids, the CLE, covering the corneocyte envelope and forming a scaffold for the lamellar lipids. *B,* our working hypothesis (8) entails LOX-catalyzed oxidation of the linoleate in EOS ceramide, facilitating hydrolysis of the ester bond, freeing OS ceramide for coupling to the corneocyte envelope protein by transglutaminase (TGase), thus forming the CLE. The electron micrograph is from Ref. 66 with permission.

Construction of the mammalian epidermal water barrier involves the coordinated actions of many gene products, the inactivating mutations of which lead to the fish skin symptoms of congenital ichthyosis in affected human families (1) and neonatal lethality in mice due to the transepidermal water loss (2). The genetic evidence identifies the enzymes 12*R*-lipoxygenase (12*R*-LOX)^4^ and epidermal lipoxygenase-3 (eLOX3) as among those critical for construction of an intact epidermal water barrier (3–7). Deletion of either enzyme results in a structural defect in the epidermal barrier, namely ∼50–99% reduction in omega-hydroxyceramide (OS) covalently bound to protein and the consequent absence or disruption of the corneocyte lipid envelope (CLE) (7, 8). The CLE is considered to be an integral component of the barrier, being a critical intermediary between polymerized protein and extracellular lamellar lipids (Fig. 1*A*) (9, 10). A marked reduction in covalently bound ceramides is also a feature of the barrier defect in essential fatty acid (EFA) deficiency (11).

**SCHEME 1.**
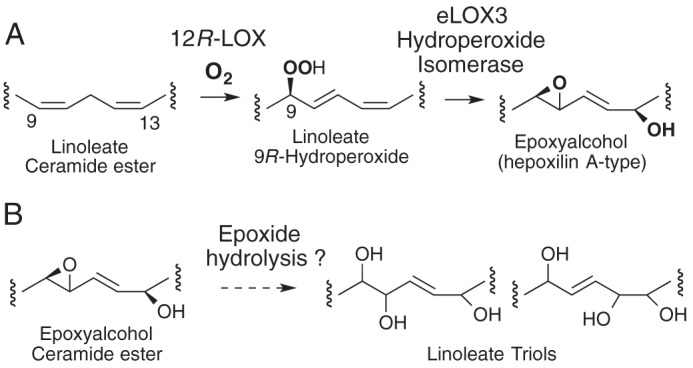
*A,* partial structures showing reaction of 12R-LOX and eLOX3 on linoleate ceramide esters. *B,* potential transformation of linoleate epoxyalcohol to triols.

Starting from the very discovery of essential fatty acids, it was recognized that linoleic acid plays a role in sealing the mammalian epidermal water barrier (12, 13). A classic symptom of EFA deficiency is transepidermal water loss with development of a scaly skin, the latter in compensation or response to the barrier defect. Topical application of linoleate restores barrier function and corrects the scaly skin phenotype (14, 15). Linoleic acid is by far the most abundant polyunsaturated fatty acid present in the mammalian outer epidermis (16). It is almost exclusively esterified in epidermis-specific ceramides, glucosyl *O-*acyl-ceramide, and its deglucosylated product, *O-*acyl-ceramide, also known as ceramide EOS, esterified omega-hydroxyacyl-sphingosine (Fig. 1*B*) (17–19). The linoleate-containing EOS ceramides are substrates for the enzymes 12*R*-LOX and eLOX3 *in vitro*, and wild-type pig and mouse epidermis contain trace quantities of specific 12*R*-LOX and eLOX3 products esterified in the EOS ceramide (Scheme 1*A*); these oxidized linoleates are absent in the 12*R*-LOX knock-out (8). Taking all the information on EFA, LOX enzymes, and the consequences of the gene knock-outs together, Zheng *et al.* (8) proposed a model to explain their cooperation in forming the epidermal barrier. The concept is that LOX activity is required to oxidize the linoleate in EOS ceramide, which in turn is required to facilitate esterase-catalyzed cleavage of the (oxidized) fatty acid, thus converting EOS to OS, the ω-hydroxyceramide used to bond to polymerized protein and thus construct the CLE (Fig. 1*B*). In EFA deficiency, oleate, which is not a LOX substrate, replaces linoleate in EOS (20, 21); therefore, the fatty acid cannot be oxidized and cannot be cleaved, leading to a reduction in the building blocks (OS ceramide) available for construction of the CLE. In the LOX knock-outs, the enzyme(s) catalyzing the oxidation are absent, with similar functional consequences.

This study, to analyze epidermis for esterified trihydroxy-linoleate species, was prompted by our difficulties in detecting the initial oxidized ceramide species in human epidermis that we had found earlier in mouse and pig skin (Scheme 1*A*) (8). We hypothesized linoleate oxidation in the epidermis might proceed further than the two steps catalyzed by 12*R*-LOX and eLOX3 and that the trihydroxy hydrolysis products of these intermediates might be the final oxidized product of the pathway (Scheme 1*B*). We therefore sought to identify and measure normal human and pig epidermis for the presence of linoleate triols derived via 12*R*-LOX/eLOX3 catalysis.

In itself the characterization of trihydroxy-linoleates is challenging. The analyses are complicated by the number of possible isomers, the symmetry of many of the triol structures, by their subtle differences in mobility in most chromatographic systems, and in some cases by the poor chromatographic performance, with the tailing of one peak into another. To circumvent these potential issues, we developed a new method applicable to fatty acid triol analysis, encompassing both improved chromatographic resolution with highly sensitive detection of the pentafluorobenzyl (PFB) ester derivative by LC-MS. In addition to the analysis of epidermal linoleate triols, the new methodology presented here has potential application to analysis of triols in plant embryogenesis and development (22), in plant defense (23, 24), and in commercial flavoring (as fatty acid triols are bitter-tasting constituents of foodstuffs and beer (25–28)). The methodology is also adaptable for analysis of the arachidonate triols implicated as an arterial endothelium-derived hyperpolarizing factor (29, 30) and the 12-LOX-derived trioxilins (31, 32).

## Experimental Procedures

### 

#### 

##### Reagents

Linoleic acid was purchased from Nu-Chek Prep Inc. (Elysian, MN); [9,10,12,13-^2^H_4_]linoleic acid was from Cayman Chemical (Ann Arbor, MI), and [1-^14^C]linoleic acid was from PerkinElmer Life Sciences Radiochemicals. 9*S*-HPODE was prepared using the 9*S*-LOX in potato homogenate as described previously (33), and 9*R*-HPODE using *Anabaena* 9*R*-LOX (34). Epoxyalcohol and triol derivatives of 9-hydroperoxy-linoleic acid were prepared as described previously (35). Ceramide-EOS was a generous gift from Evonik. Pentafluorobenzyl bromide (PFBBr), *p*-toluene sulfonic acid, pyridinium *p*-toluenesulfonate, diisopropylethylamine, 2,2-dimethoxypropane (DMP), trimethylamine, and *N,O*-bis(trimethylsilyl)trifluoroacetamide were obtained from (Sigma).

##### Preparation of a Mix of Eight Deuterated Triols by Autoxidation of [^2^H_4_]Linoleic Acid

Deuterated linoleic acid (5 mg) was methylated with diazomethane, dissolved in ethanol (EtOH) with 10% α-tocopherol, transferred to a 20-ml vial and taken to dryness, and then left in an atmosphere of oxygen for 1 week at room temperature. The autoxidized sample was subsequently hydrolyzed to the free acids by addition of 2 ml of methanol (MeOH), 200 μl of dichloromethane (DCM), and 2 ml of 1 m KOH and incubated for 40 min at room temperature. The solution was acidified to pH 5 and extracted by vigorous mixing with 3 ml of ice-cold DCM; the organic phase was washed twice with ice-cold water, transferred to a fresh vial, dried under N_2_, and dissolved in 25 μl of MeOH. Five ml of 0.1 m K_2_HPO_4_ and 200 μl of hematin (10 mg/ml in DMSO) were added and left to react for 15 min at room temperature. To hydrolyze the resulting allylic epoxides to triols, the sample was acidified to pH 3 and left standing for 1 h at room temperature. The sample was then cooled on ice and extracted with 5 ml of ethyl acetate, which was washed twice with cold water, transferred to a fresh vial, dried under N_2_, and dissolved in 1.25 ml of ethyl acetate. Hexane (3.75 ml) was added immediately before loading onto a 0.5 g of Bond-Elut silica cartridge pre-conditioned with 25% ethyl acetate in hexane. After washing the column with an additional 10 ml of 25% ethyl acetate in hexane and 10 ml of 100% ethyl acetate, the triol products were eluted with 5% MeOH in ethyl acetate (10 ml). The *d*4-triols were subsequently purified as a group by reversed phase-HPLC employing a Waters Symmetry C18 column (250 × 4.6 mm) eluted isocratically with methanol/water/acetic acid (70:30:0.01) at 1 ml/min. The 205-nm absorbing peaks in the triol region of the chromatogram at 8–10 min of retention time were collected and used without further purification. This mixture of *d*4-triols was difficult to quantify because of the absence of weighed triol standards for comparison and also the multiple overlapping peaks on reversed phase-HPLC and SP-HPLC chromatograms. The yield was estimated as ∼100 μg of the *d*4-triol mixture.

##### Preparation of Deuterated 9-HODE, Epoxyalcohol, and Triols from [^2^H_4_,^14^C]Linoleic Acid

Deuterated linoleic acid (1 mg, 3.57 μmol) was mixed with [1-^14^C]linoleic acid (1 μCi), and a small aliquot was counted. Subsequent quantitation of *d*4-products was based on the known ^14^C-specific activity (which for the conjugated diene-containing products 9-HPODE and 9-HODE was checked by UV spectroscopy). The [^2^H_4_,^14^C]linoleic acid was reacted with *Anabaena* 9*R*-LOX (34), and the 9*R*-HPODE product was purified by SP-HPLC and quantified by UV spectroscopy, and an aliquot was counted. The 9*R*-HPODE (0.7 mg) was then reacted with hematin as above, and the epoxyalcohols and other products were separated by SP-HPLC (35); aliquots of the ^2^H_4_/^14^C-labeled 9-HODE and 9*R*,10*R-trans*-epoxy-13*R*-hydroxy epoxyalcohol were quantified by liquid scintillation counting. The deuterated 9-HODE and one-third of the *d*4-epoxyalcohol were kept for use in quantitative assays. The remainder of the epoxyalcohol was treated with 200 μl of 1% acetic acid in CH_3_CN/H_2_O (1:1 v/v) for 1 h at room temperature, then the solvent was evaporated using a stream of nitrogen. The resulting ^2^H_4_/^14^C-labeled triol isomers were partially resolved at long retention times (∼120 min) on a Waters symmetry column (25 × 0.46 cm) using a solvent of CH_3_CN/H_2_O/HAc (30:70:0.01 v/v/v) run at 0.5 ml/min with UV detection at 205 nm. A fraction containing a mixture of mainly triols 3 and 4 (as assigned later) in equal proportions was identified by LC-MS and quantified by liquid scintillation counting. (The fraction containing *d*4-triols 3 and 4 was not further purified because of the limited quantity available and the lack of a suitable chromatographic system for their separation.) For quantitative analysis of triol- 3 in epidermis, the samples were spiked with 0.2 nmol of the mixture (and thus 0.1 nmol of *d*4-triol-3 added).

##### Preparation of Epidermis

Pig skin was obtained within 30 min of euthanasia from animals used in unrelated experiments approved by the Institutional Animal Care and Use Committee. Sections of pig skin (∼10 × 10 cm) were immersed in 65 °C phosphate-buffered saline (PBS) for 2 min, and then the epidermis was separated from the dermis using tweezers. Normal human skin was provided by healthy donors (female; 29–53 years old; African American and Caucasian), undergoing abdominoplastic surgery, and delivered to our laboratory within 1 h of the operation. Informed consent was obtained from each patient, and the clinical research ethics committee of Vanderbilt University reviewed and approved the study. The human epidermis (∼10 × 10 cm) was isolated after treating with 2.0 mg/ml Dispase II (Roche Applied Science) in 50 mm HEPES, pH 7.4, at 4 °C overnight.

##### Lipid Extraction

The epidermis was blotted dry, weighed, then homogenized in chloroform/methanol mixtures (1:1, v/v). The organic layer was separated from the protein pellet by centrifugation. This was repeated three times. The lipid extract was dried under a stream of N_2_ and then redissolved in chloroform. The lipid extract was loaded onto a pre-equilibrated solid phase silica column in chloroform/hexane (1:1, v/v) (HF Bond Elut SI, Agilent), washed with chloroform/hexane (1:1, v/v), and eluted with chloroform and chloroform/methanol (9:1, v/v); the fractions were taken to dryness, and each was redissolved in 1 ml of CHCl_3_ for further analysis.

##### LC-MS Screening of Epidermal Extracts for Linoleate and Linoleate Triol-containing Ceramides

Methanol/CHCl_3_ extracts were analyzed using an Advantage 5-μm silica column (250 × 4.6 mm) run with gradient elution of hexane/IPA/HAc (95:5:0.1) to hexane/IPA/HAc (75:25:0.1) over 30 min using a Waters Alliance 2690 HPLC system coupled to a TSQ Vantage mass spectrometer (Thermo Scientific) with instrument conditions as reported previously (8). The spectra were obtained in full scan mode between the mass range *m/z* 650–1350 monitoring in +ve APCI mode. SIM was carried out in the same system in -ve APCI mode to allow detection of the daughter free fatty acids.

##### Identification of Free and Esterified Triols in Epidermis

For analysis of the linoleate triol isomeric composition, aliquots of the combined CHCl_3_ and 10% MeOH in CHCl_3_ eluates (corresponding to ∼10 mg of epidermis extracted) containing free and esterified fatty acid triols were spiked with the *d*4-linoleate triol mixture (∼40 ng) and processed in one of three ways (illustrated in supplemental Fig. S1). For analysis of free triols, the sample is initially subjected to a Bligh and Dyer extraction using pH 8 aqueous phase; the ionized trihydroxy fatty acids remain in the aqueous phase while the neutral and non-polar lipids partition into the CHCl_3_ phase and are discarded; extraction with theoretical lower phase is repeated and the CHCl_3_ again discarded. After evaporation of the aqueous phase to half volume to remove residual CHCl_3_ and most of the MeOH, the sample is diluted with water, acidified to pH 6 (mildly acidic, to avoid acid hydrolysis of epoxyalcohols), and loaded onto a 1-cc/30-mg Oasis HLB cartridge (Waters). After elution of salts and aqueous-soluble impurities using water/MeOH (9:1), the triols are recovered by elution with water/MeOH (1:9). For analysis of esterified triols, the samples from the initial silica cartridge fractionation were treated with KOH overnight to release esterified fatty acids (total volume of 500 μl consisting of 100 μl of 10% MeOH in CHCl_3_ epidermal extract, 275 μl of MeOH, and 125 μl of 2 m KOH in 20% water in MeOH, final KOH concentration, 0.5 m). The de-esterified products were then extracted using the Bligh and Dyer proportions of CHCl_3_, MeOH, and aqueous phase (still containing the KOH) with the CHCl_3_ phase discarded; extraction was repeated using theoretical lower phase. After concentration of the aqueous phase to about half-volume, the sample is diluted with water and carefully acidified to pH 6 for Oasis HLB extraction as for the free triols, or alternatively, the sample is acidified to pH 3 and allowed to stand at room temperature for 1 h (to permit acid hydrolysis of labile epoxyalcohols) and then extracted on the Oasis HLB cartridge. All samples are converted to the PFB ester DMP acetonide derivative for LC-MS, GC-MS, or chiral HPLC-UV analysis. For GC-MS and chiral HPLC analyses of individual triols, the scheme was repeated using larger aliquots of epidermis extract and without addition of the *d*4-triol mixture.

##### Quantitation of 9-HODE, Epoxyalcohol, and Triols in the Epidermis

To optimize recovery of the acid-sensitive epoxyalcohol, the extracts were kept neutral or alkaline throughout the analytical procedure. The scheme is summarized in supplemental Fig. S2. Aliquots (2%, corresponding to an extract of ∼10 mg of epidermis) of the combined CHCl_3_ and 10% MeOH/CHCl_3_ fractions from the open-bed silica column (and for free triol analysis also combined with the MeOH silica column eluate) were spiked with 0.1 nmol of *d*4–9-HODE, 0.1 nmol of the *d*4-epoxyalcohol (9*R*,10*R-trans*-epoxy-13*R*-hydroxy-octadec-11*E*-enoic acid), and 0.2 nmol of the *d*4-triol (composed of equal proportions of triols-3 and -4). For analysis of esterified products, the samples were treated with KOH overnight as described above and then extracted twice using the Bligh and Dyer proportions of CHCl_3_, MeOH, and (KOH) aqueous phase. After concentration of the aqueous phase (still containing the KOH) to approximately one-third volume (∼0.5 ml) and addition of 2 ml of water, the alkaline solution was loaded onto a 1-cc/30-mg Oasis HLB cartridge that was pre-equilibrated with MeOH then water; 2 ml of water, the alkaline solution was loaded onto a 1 ml/30 mg of Oasis HLB cartridge that was pre-equilibrated with MeOH then water; after washing with water until a neutral reaction, the products were eluted with 1 ml of EtOAc. The free product levels were quantified in essentially the same way except omitting the KOH hydrolysis and substituting 0.1 m K_2_HPO_4_, pH ∼8.5, for the Bligh and Dyer extraction and HLB extraction steps. Although the samples were loaded onto the HLB cartridge in alkaline solution, recoveries of the linoleate derivatives were quantitative.

##### Derivatization Procedures

PFB esters were prepared by dissolving the *d*4 standards or human skin extracts in 20 μl of acetonitrile, 20 μl of PFBBr in acetonitrile (1:19, v/v), and 20 μl of diisopropylethylamine in acetonitrile (1:9, v/v). The solution was incubated at room temperature under argon for 30 min and then evaporated to dryness under nitrogen. Acetonide derivatives of the triols were prepared by addition of DMP/acetone (1:1) containing 1 mm *p*-toluene sulfonic acid to the dry PFBBr ester derivatives (reaction was complete within 5 min at room temperature); the acid was then neutralized using an equal volume of triethylamine (1.5 mm) in DCM, and the sample was taken to dryness. Alternatively, the DMP derivative was prepared using 20 μl of 1 mm pyridinium *p*-toluenesulfonate in acetone/DMP (1:1 by volume) for 30 min at room temperature and then directly taken to dryness (as neutralization is not required using the pyridinium salt).

##### LC-MS Analyses

PFB ester DMP derivatives of the linoleate triols were detected by APCI-LC-MS using a TSQ Vantage instrument (Thermo Scientific), Waters Alliance 2690 HPLC system, and a Thomson Advantage 150A 5-μm silica column (250 × 4.6 mm) with a solvent of hexane/IPA (100:1, v/v) and a flow rate of 1 ml/min. The APCI vaporizer temperature was set to 300 °C, and the heated electrospray ionization probe temperature was set to 150 °C with selected ion monitoring of negative ions at *m*/*z* 369 for the unlabeled triol derivatives and *m*/*z* 373 for the *d*4 internal standard. Quantitation of 9-HODE used the same equipment with an SP-HPLC solvent of hexane/IPA (100:0.5, v/v) and monitoring *m*/*z* 295 and 299. For epoxyalcohol quantitation the SP-HPLC solvent was changed to hexane/IPA (100:1.5, v/v), and the ions monitored were *m*/*z* 311 and 315.

##### GC-MS

Analysis of the major epidermal triol was carried out on the PFB ester DMP acetonide and trimethylsilyl ether derivative using an Agilent/J and W DB-5MS column (25 m × 0.2 mm, 0.33-μm film) in an Agilent 6890 GC/5973 MSD instrument operated in the electron ionization mode (ion source temperature 250 °C, electron energy 70 eV) set to full scans from *m*/*z* 50 to 700.

##### Chiral Analysis of Epidermal Products

Chiral HPLC analysis of linoleate triols was conducted on the PFB ester DMP derivatives using a Chiralpak 5-μm AD-H column, 150 × 2.1 mm (Chiral Technologies, Exton, PA), a solvent of hexane/MeOH (100:2), and a flow rate of 0.25 ml/min, with LC-MS monitoring of *m*/*z* 369 in the APCI mode as described above. Racemic triol standards were prepared from racemic 9-HPODE and standards of known chirality from 9*S*-HPODE using the methods previously described (35). There was sufficient of the main epidermal triol of pig epidermis that the chiral HPLC analysis did not require use of LC-MS for sensitivity; the analysis was preformed with UV detection of the PFB DMP derivative at 205 nm.

For chiral analysis of 9-HODE and the epoxyalcohol 9,10-*trans*-epoxy-13-hydroxy-octadec-11*E*-enoic acid esterified in human epidermis, 20% of extracts of 10 × 10 cm pieces of epidermis were taken through the extraction procedure outlined above (except with no added *d*4 standards and using a 4-fold increase in solvent volumes for the KOH hydrolysis and Bligh and Dyer extraction). After application of ∼3 ml of KOH solution to the 30-mg Oasis HLB cartridge, elution with EtOAc (1 ml), and subsequent preparation of the PFB esters, samples were subjected to SP-HPLC using a Thomson silica column with a solvent of hexane/IPA (100:1.5 v/v) run at 0.5 ml/min with UV monitoring at 205, 220, 235, and 270 nm and simultaneous recording of UV spectra. The easily visible peak of 9-HODE (attaining a peak absorbance of ∼300–400 mAU and preceded by equally prominent peaks of 12-HETE and 13-HODE PFB esters) was collected at an ∼10-min retention time, and the single prominent peak of epoxyalcohol PFB ester (visible most prominently at 205 nm) was collected at ∼16 min retention time. Chiral analysis of the purified 9-HODE PFB ester used a Chiralcel OD-H column (25 × 0.46 cm), a solvent of hexane/IPA (100:5 v/v), and a flow rate of 1 ml/min; the enantiomers eluted at 9.1 min (9*S*) and 10.5 min (9*R*) and were detected by UV monitoring at 235 nm. Chiral analysis of the purified PFB ester of the epoxyalcohol (9,10-*trans*-epoxy-13-hydroxy-octadec-11*E*-enoic acid) from human skin employed a Chiralcel OJ column (25 × 0.46 cm) with a solvent of hexane/IPA 100:10 (v/v) and a flow rate of 1 ml/min with LC-MS (APCI) monitoring of *m*/*z* 369; the enantiomers eluted at 7.6 (9*S*,10*S*,13*S* configuration) and 8.8 min (9*R*,10*R*,13*R*) as established using epoxyalcohol standards prepared from 9*RS*-HPODE and 9*S*-HPODE.

##### Quantitative Analysis of Esterified Arachidonic Acid (AA), Linoleic Acid (LA), and α-Linolenic Acid (α-LNA) in the Epidermis

The esterified AA, LA, and α-LNA in the epidermal ceramide fractions from the initial silica column were analyzed using *d*4-LA as internal standard: 1% percent (10 μl from 1 ml) of the 10% MeOH in CHCl_3_ and CHCl_3_ fractions in 1.5-ml Eppendorf tubes was treated with 100 μl of 1 m KOH in 20% water in MeOH under argon at room temperature overnight. *d*4-LA (10 μl, 10 μg) was added, and then the samples were acidified to pH 4 by addition of 625 μl of 1 m acetic acid, and the free fatty acids were extracted with 0.75 ml of 10% ethyl acetate in hexane. The solution was taken to dryness and converted to the PFB ester for LC-MS analysis; the PFB esters were dissolved in 1 ml of hexane and 1% injected. PFB esters of AA, LA, and α-LNA in the epidermis were detected using the TSQ Vantage instrument coupled to a Waters Acquity UPLC system with a Phenomenex Kinetex 2.6 -μm C18 column (100 × 3 mm), a solvent of MeOH/H_2_O/HAc (95:5:0.01, v/v/v), and a flow rate of 0.5 ml/min. Negative ions were monitored at *m*/*z* 277 (for α-LNA), *m*/*z* 279 (LA), *m*/*z* 283 (*d*4-LA), and *m*/*z* 303 (AA).

## Results

### 

#### 

##### LC-MS Detection of Epidermal Ceramides Containing Linoleate Triols

**FIGURE 2.**
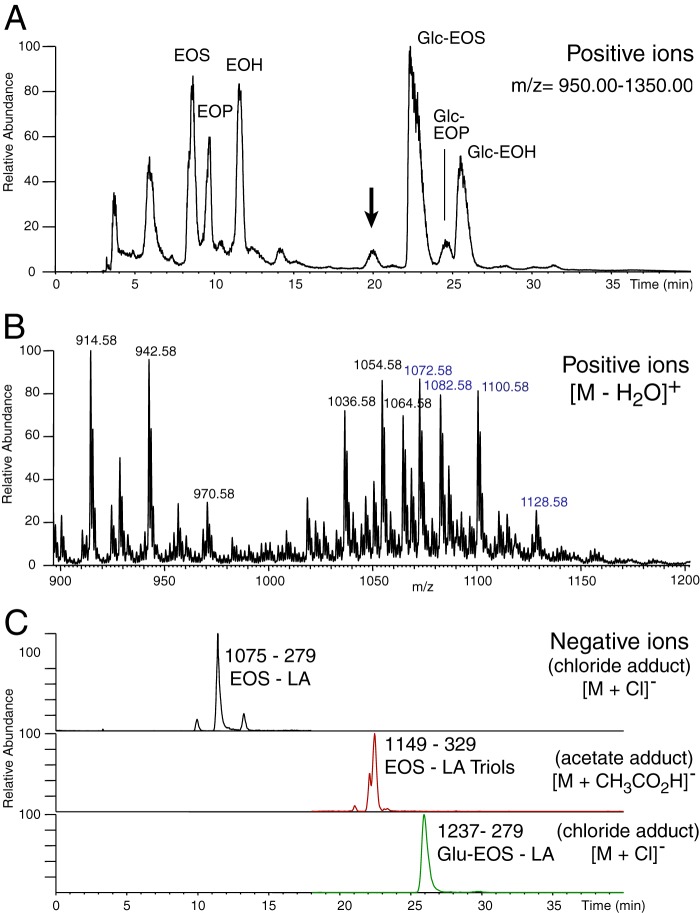
**LC-MS analysis of human epidermal extracts for linoleate and linoleate triol-containing ceramides.** *A,* total ion current profile (*m*/*z* 950–1350) of LC-MS analysis of a MeOH/CHCl_3_ extract of human epidermis using an Advantage 5-μm silica column (250 × 4.6 mm) run with gradient elution of hexane/IPA/HAc (95:5:0.1) to hexane/IPA/HAc (75:25:0.1) over 30 min using a Waters Alliance 2690 HPLC system coupled to a TSQ Vantage mass spectrometer (Thermo Scientific). The *arrow* at 20 min marks the retention time of putative linoleate triol-containing ceramides. *B,* extracted ion chromatogram of the molecular species eluting at 20 min. *C,* selected ion monitoring of C32 ceramide species to the respective daughter ions of *m/z* 279 (linoleate) and *m/z* 329 (linoleate triols), confirming identification of EOS (*m/z* 1075–279 [M + Cl]-), EOS-triol (*m/z* 1149–329 [M − H + CH_3_CO_2_H]-), and Glu-EOS (*m/z* 1237–279 [M + Cl]-).

Human epidermal extracts were screened for the possible occurrence of oxidized ceramides using straight-phase HPLC separation of the ceramide esters with LC-MS detection of either positive or negative ions. Fig. 2*A* illustrates an extracted ion chromatogram of positive ions in the mass range *m*/*z* 950–1350. This mass range encompasses the acyl-ceramides (EOS-related) and excludes lower weight species such as typical ceramides and phospholipids. The ceramides with unoxidized fatty acid esters eluted early in this SP-HPLC system and can be identified as EOS, EOP, and EOH. The polar glucosyl ceramides eluted late in the chromatogram and can be identified as Glc-EOS, Glc-EOP, and Glc-EOH (data not shown). Fig. 2*B* shows the mass spectrum of the peak of interest eluting at 20 min; the masses correspond primarily to ceramides with C32 and C34 *N*-linked very long chain fatty acids coupled by ester linkage to esterified trihydroxy-linoleates. In-source fragmentation results in several characteristic ions, for example *m/z* 1054 and 1072 represent the [M − 2H_2_O]^+^ and [M − H_2_O]^+^ ions of the C32 species, and *m/z* 1082 and 1100 represent the [M − 2H_2_O]^+^ and [M − H_2_O]^+^ of the C34 ceramide. To confirm the structural assignment of these ceramides, an SIM method was developed to monitor the specific parent to daughter transition for the C32 carbon molecule of each species. Fig. 2*C*, recorded in the negative ion mode for fatty acid detection, illustrates the SIM chromatogram for the following species: C32 EOS, *m/z* 1075–279 [M + Cl]^−^, C32 EOS-tri-OH, *m/z* 1149–329 [M − H + CH_3_CO_2_H]^−^, and *m/z* 1237–279 [M + Cl]^−^, with *m*/*z* 279 and 329 confirming the presence of linoleate and linoleate triol, respectively. Very similar results were obtained in analysis of porcine epidermis (data not shown).

##### LC-MS Analysis of Linoleate Triols as the PFB Ester DMP Derivative

**FIGURE 3.**
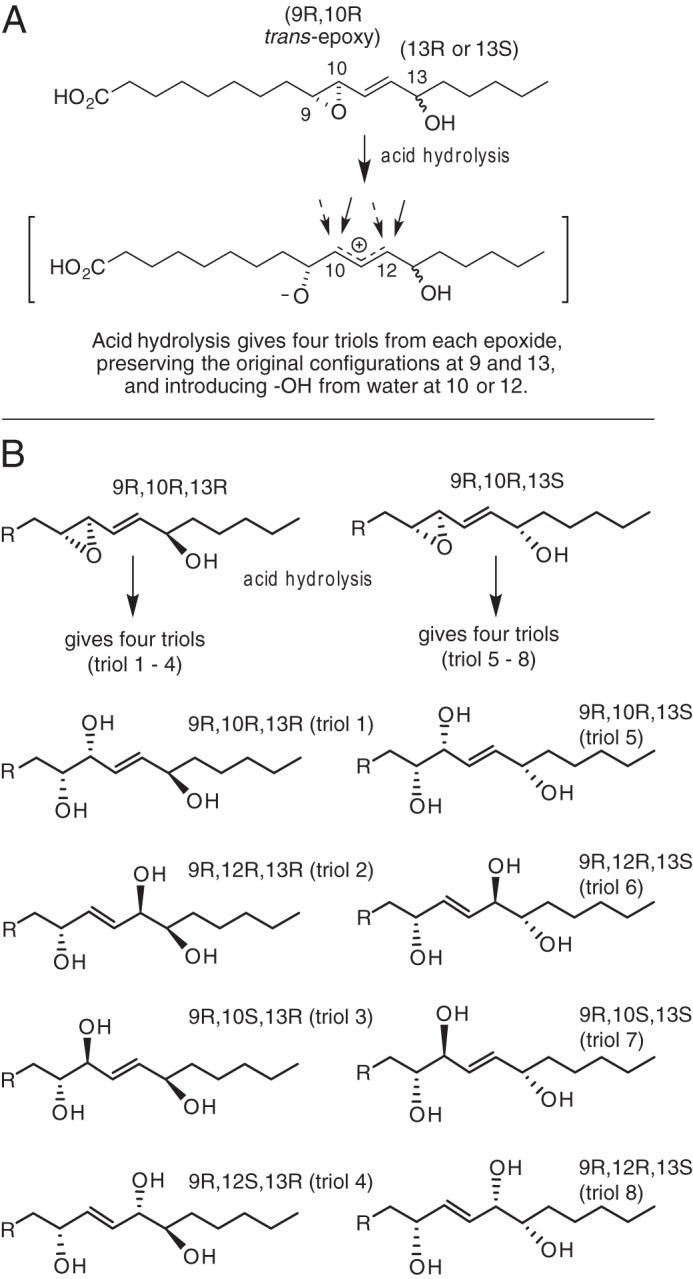
**Acid hydrolysis of linoleate 9,10-epoxy-13-hydroxy epoxyalcohols and structures of the resulting triols.** *A,* acid hydrolysis opens the epoxide ring and permits reaction of water at C-10 or C-12, producing four triol products from a single epoxyalcohol. *B, left side*, hydrolysis of 9*R*,10*R-trans*-epoxy-11*E*-13*R*-hydroxy-octadecenoate produces triols 1–4. *Right side*, hydrolysis of the diastereomer with 13*S*-hydroxyl produces triols 5–8. Not illustrated are the eight enantiomeric triols arising from the mirror image epoxyalcohols of 9*S*,10*S*-epoxy-13*RS*-hydroxy configuration. Authentic samples of triols 1–8 and the enantiomers were prepared as standards for comparison with the epidermal triols.

Having detected epidermal ceramide species containing esterified linoleate triols, we developed methods for the analysis and quantitation of individual trihydroxy isomers. As outlined under “Experimental Procedures,” the authentic trihydroxy-linoleate standards were prepared by hematin treatment of 9-HPODE followed by separation of the two major 9,10-*trans*-epoxy-13-hydroxy epoxyalcohol diastereomers and their acid hydrolysis to give four triols from each (Fig. 3*A*) (35). The first of the two epoxyalcohols to elute from SP-HPLC, 9*R*,10*R*-epoxy-13*R*-hydroxy-octadec-11*E*-enoic acid, is hydrolyzed under mild acidic conditions to the four triols designated here as 1–4, and the second epoxyalcohol diastereomer (9*R*,10*R*-epoxy-*13S*-hydroxy) gives triols 5–8 (Fig. 3*B*). The structure and stereochemistry of all these products were assigned partly from the known configuration of the 9-HPODE starting material. The epoxyalcohols were then characterized by mass spectrometry and proton NMR, plus circular dichroism to establish the configuration of the 13-hydroxyl, although the individual triol structures were assigned based on the structure of their parent epoxyalcohol together with GC-MS and proton NMR analyses (35).

**FIGURE 4.**
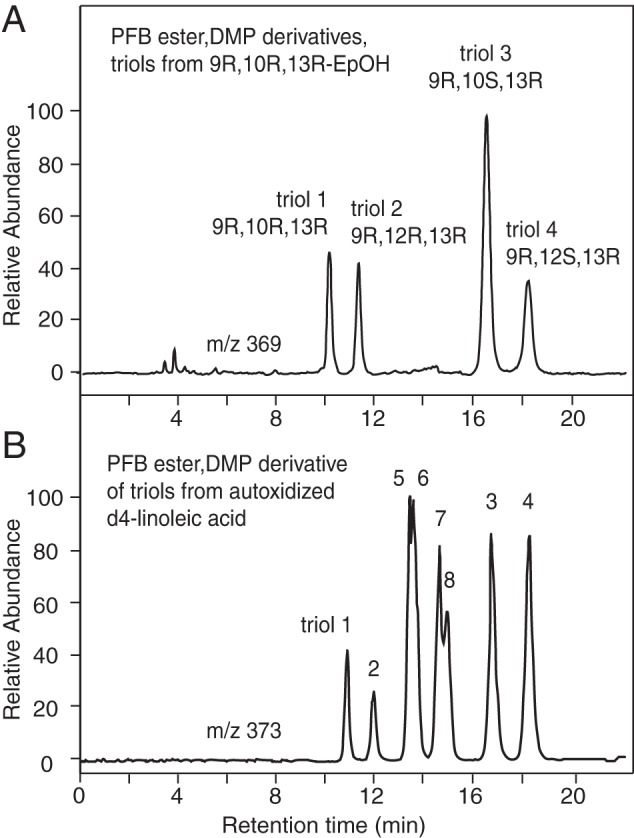
**LC-MS resolution of linoleate triol standards as the PFB ester DMP derivative.** *A,* four triol hydrolysis products (triol-1 to -4) of 9*R*,10*R*-epoxy-13*R*-hydroxyoctadec-11*E*-enoic acid are analyzed as the PFB ester DMP derivative using a Thomson Advantage Silica 150Å 5-μ, column (25 × 0.46 cm) with a solvent of hexane/IPA 100/1 (by volume) at a flow rate 1 ml/min, with UV detection at 205 nm. *B,* in autoxidized *d*4 epoxy alcohol, the same four products were chromatographed with the triols (PFB ester DMP derivative) from the second major epoxy alcohol of the hematin reaction, the 13*S* diastereomer, 9*R*,10*R*-epoxy-13*S*-hydroxyoctadec-11*E*-enoate (triol-5 to -8).

To develop a method capable of resolving this complex mixture of linoleate triols and to permit analysis by LC-MS, we adapted our recently reported procedure employing SP-HPLC of the methyl ester DMP acetonide derivative (35). By substituting use of the PFB ester in place of the methyl ester, the analyte becomes amenable to sensitive detection by negative ion/chemical ionization mass spectrometry (Fig. 4) (36, 37). The PFB esters are less polar than the corresponding methyl esters and elute slightly earlier on SP-HPLC, but otherwise the separations are comparable. Fig. 4*A* illustrates separation of the PFB-DMP derivative of triols 1–4 derived from the 9*R*,10*R*,13*R* epoxyalcohol and detected by LC-MS monitoring of the M-PFB ion at *m*/*z* 369. The corresponding chromatogram of all eight triols are shown in Fig. 4*B*, this mixture being the *d*4-triols we prepared from *d*4-linoleic acid (see “Experimental Procedures”) and detected at 4 atomic mass units higher at *m*/*z* 373.

##### Identification of a Major Esterified Linoleate Triol in Pig Epidermis

**FIGURE 5.**
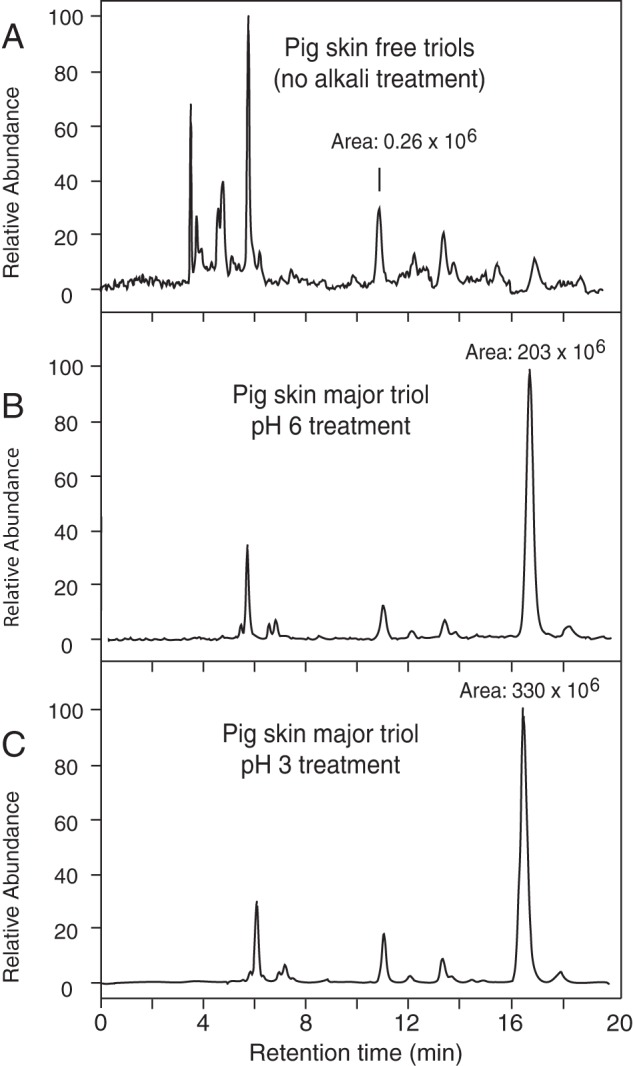
**LC-MS analysis of triols from pig epidermis.** SIM chromatograms (*m*/*z* 369) of pig epidermal triols analyzed by normal-phase LC-APCI-MS. *A,* analysis of free triols (non-alkali treated sample); *B,* esterified triols, extracted at pH 6; *C,* esterified triol analysis, pH 3-treated. Intensity was normalized to the level in *C*. Analysis of the back skin of three animals gave a very similar pattern of esterified triols with the same prominent peak of triol-3 (Table 1).

**FIGURE 6.**
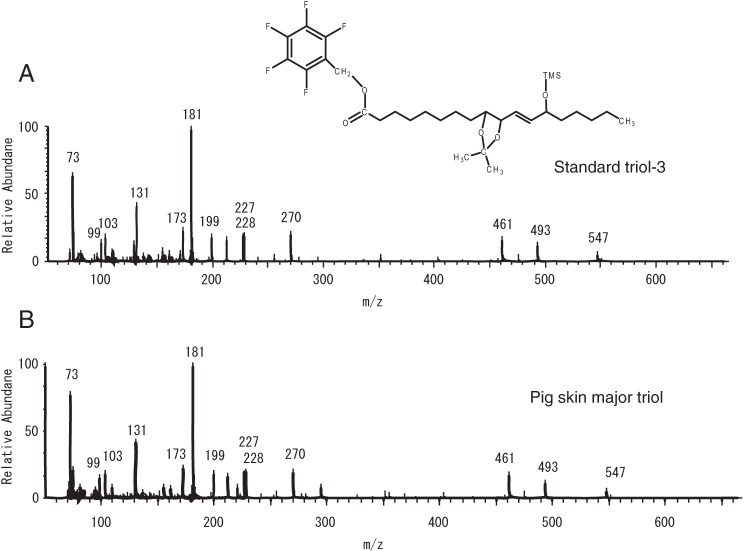
**GC-MC analysis of the major pig epidermis triol.** *A,* electron ionization mass spectrum of authentic triol-3 as the PFB ester, DMP acetonide, trimethylsilyl ether derivative. *B,* major esterified triol from pig skin was collected from SP-HPLC as the PFB DMP derivative, converted to the TMS ester derivative, and analyzed by GC-MS, exhibiting the same GC retention time and essentially identical mass spectrum to the authentic standard.

Initial LC-MS analyses of extracts of pig epidermis taken through the extraction procedure revealed only trace quantities of free triols (<1% of the amounts esterified, Fig. 5*A*) and a particularly prominent peak at ∼16.5 min among the esterified triols (Fig. 5, *B* and *C*). As pH 3 treatment of any epoxyalcohols present in the extracts would form a nonspecific mixture of triols and as the profiles in Fig. 5, *B* and *C,* are essentially indistinguishable, this suggests that non-enzymatic epoxyalcohol hydrolysis contributed little toward these measurements. Although the prominent peak at 16.5 min co-chromatographed with triol-3 of our standards, we considered that further proof of its structure was required. Accordingly, first we recorded full scans (*m*/*z* 100–700) on the esterified samples, proving that all peaks corresponding to the triol standards showed essentially indistinguishable NICI mass spectra with a major fragment ion at *m*/*z* 369 due to loss of the PFB ester (data not shown). There was sufficient of the major peak from pig epidermis to allow its detection by HPLC with UV detection and its UV spectrum matched that of a PFB ester. This peak was collected, and after silylation on the free hydroxyl with *N,O*-bis(trimethylsilyl)trifluoroacetamide, it was analyzed by GC-MS in the electron impact mode (Fig. 6). The major triol from pig epidermis had the same retention time on GC as the PFB ester DMP TMS ether derivative of the triol-3 standard. The mass spectra of sample and standard were essentially identical with diagnostic ions at *m*/*z* values of 547 (M − 129), 493 (M − 147), 461 (M − [71 + Me_3_SiOH]), 270 (Me_3_SiO^+^ − PFB), 227 and 228, 199 (C11 to C18), 181 (PFB), 173 (C13 to C18), 99 (C13 to C18 − Me_3_Si^+^), and 73 (Me_3_Si^+^), thus confirming the structure as a 9,10,13-trihydroxy-octadecenoate. The mass spectrum is similar in character and shows the corresponding fragmentation to the published spectrum of the corresponding methyl ester derivative (35).

**FIGURE 7.**
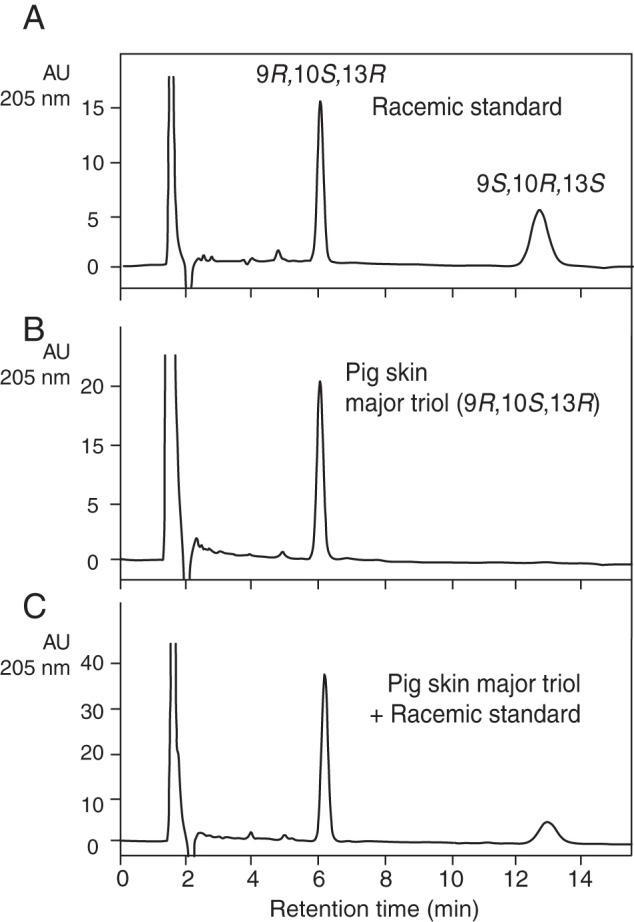
**Chiral HPLC of pig epidermis triol.** The analyses were conducted using a Chiralpak AD-H column (100 × 2.1 mm), a solvent of hexane/MeOH (100:2 v/v) at a flow rate of 0.25 ml/min with UV detection as 205 nm. *A,* racemic standard of triol-3 PFB-DMP derivative. *B,* pig epidermal major triol. *C,* co-injection of racemic standard of triol-3 PFB-DMP and the pig epidermal major triol. *AU*, absorbance units.

Finally, using a racemic triol-3 standard prepared from 9*RS*-HPODE and a chiral triol-3 standard prepared from 9*S*-HPODE, we developed a chiral column HPLC-UV method for analysis of the stereochemistry of triol-3 as the PFB DMP derivative (Fig. 7*A*). The triol-3 from pig epidermis eluted as a single peak from the chiral column (Fig. 7*B*), and when mixed with racemic standard, it precisely co-chromatographed with the first triol-3 enantiomer (Fig. 7*C*). Thus, the prominent esterified triol of pig epidermis was unambiguously identified as 9*R*,10*S*,13*R*-trihydroxy-octadec-11*E*-enoate.

##### Identification of the Major Esterified Linoleate Triols in Human Epidermis

**FIGURE 8.**
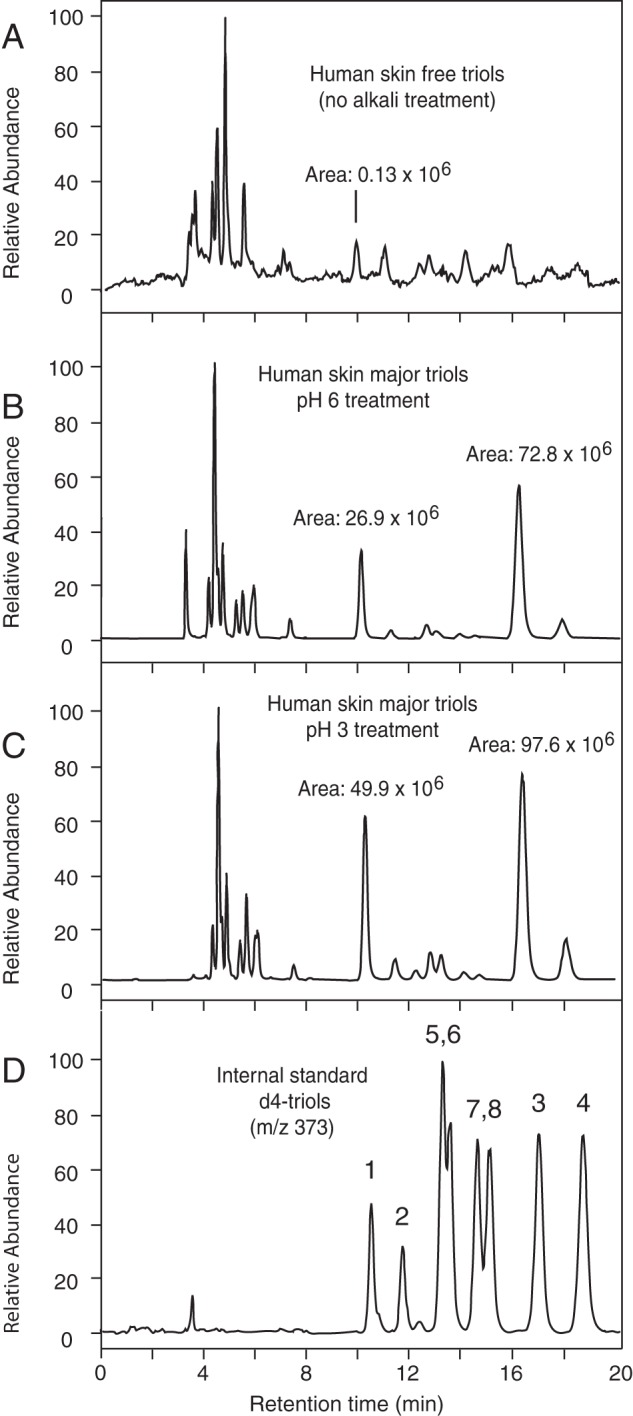
**LC-MS analysis of triols from human epidermis.** SIM profiles of the human epidermal triols (*A–C, m*/*z* 369) analyzed by normal phase LC-APCI-MS along with the *d*4-triol standards (*D*, *m*/*z* 373). *A,* analysis of free triols (non-alkali treated sample); *B,* esterified triols, extracted at pH 6; *C,* esterified triol analysis, pH 3-treated; *D, d*4-triol internal standards. The skin of three subjects was analyzed, each giving a very similar pattern of esterified triols with the same prominent peaks of triol-1 and triol-3 (Table 2).

**FIGURE 9.**
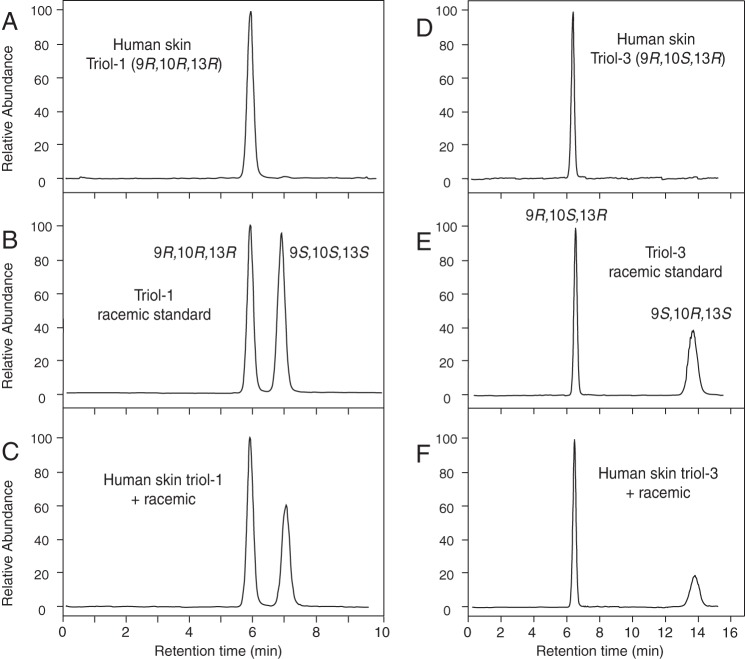
**Chiral LC-MS of human epidermis triols.** Samples were analyzed using a Chiralpak AD-H column as described in the legend to Fig. 8 except that the products were detected by APCI-LC-MS. *A,* human epidermal triol-1 PFB-DMP derivative. *B,* racemic standard of triol-1. *C,* co-injection of racemic standard of triol-1 PFB-DMP and human epidermal triol-1. *D,* human epidermal major triol-3. *E,* racemic standard. *F,* co-injection of racemic standard of triol-3 PFB-DMP and human epidermal triol-3.

Similar to pig epidermis, there are only trace levels of free fatty acid triol in human epidermis (Fig. 8*A*). The epidermis treated with pH 3 or pH 6 gave very similar profiles with major peaks at ∼8 min retention time corresponding to 9*S*,10*S*,13*S*-triol (triol-1) and the more prominent peak of 9*S*,10*R*,13*S*-triol (triol-3) eluting at ∼16 min (Fig. 8, *B* and *C*). Using the methods we developed for steric analysis of the 9,10,13-hydroxyl configuration, we identified the human epidermal triols as exclusively the 9*R*,10*R*,13*R* enantiomer of triol-1 (Fig. 9, *A–C*), and the 9*R*,10*S*,13*R* enantiomer of triol-3 (Fig. 9, *D–F*). The relative proportions of the linoleate triols 1, 2, 3, and 4 esterified in human epidermis averaged 33, <1, 63, and 3%, respectively, in three analyses.

##### Quantification of 9-HODE, Epoxyalcohol, Triols, and Fatty Acids in Porcine and Human Epidermis

**FIGURE 10.**
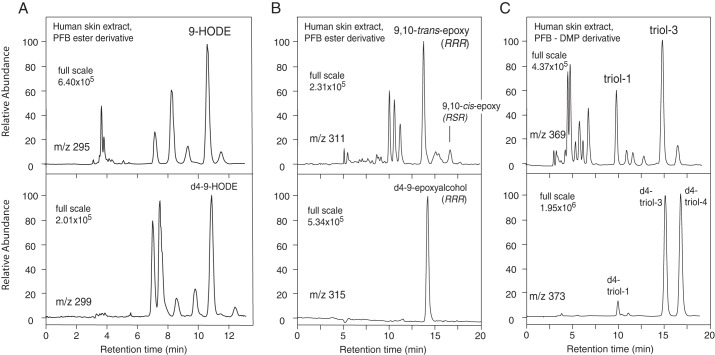
**Quantitative analyses of 9-HODE, epoxyalcohol, and triols in human epidermis.** The unlabeled products (endogenous) are in the *upper chromatograms* and the corresponding *d*4 internal standards are in the *bottom chromatograms. A,* LC-MS analysis of 9-HODE as the PFB ester, monitoring *m*/*z* 295 (*d*0) and *m*/*z* 299 (*d*4). *B,* LC-MS analysis of the main epoxyalcohol as the PFB ester (*d*0 at *m*/*z* 311 and *d*4 at *m*/*z* 315). *C,* LC-MS analysis of linoleate triols as the PFB ester DMP derivative (*d*0 at *m*/*z* 369 and *d*4 at *m*/*z* 373). The analyses used normal phase LC-MS with a Thomson Advantage silica column (25 × 0.46 cm) and solvents of hexane/IPA in the proportions 100:1.5 (v/v) for 9-HODE and epoxyalcohol analyses and 100:1 (v/v) for the triols with a flow rate in 1 ml/min.

The content of the three oxidation products quantified in porcine and human epidermis are given in Tables 1 and 2, respectively. Porcine epidermis contained 5–10-fold higher amounts of esterified epoxyalcohol and triol-3 compared with human epidermis, along with quantitatively similar levels of 9-HODE. Representative chromatograms from the quantitative analyses of human epidermal esterified 9-HODE, epoxyalcohol, and triol-3 are illustrated in Fig. 10.

**TABLE 1:** **Quantitative analysis of epidermal 9-HODE, 9-epoxyalcohol, and triol-3 in pig epidermis**

Epidermal content (per g wet weight of epidermis)	9-HODE	9,10-*trans*-Epoxyalcohol*^a^*	Triol-3
nmol/g, *n* = 3 (range)	43.7 (39.1–46.0)	23.4 (21.0–27.7)	32.7 (30.6–34.4)
μg/g, *n* = 3 (range)	13.1 (11.5–14.2)	7.3 (6.6–8.6)	10.8 (10.1–11.3)

*^a^* The epoxyalcohol assayed is 9*R*,10*R-trans*-epoxy-13*R*-hydroxy-octadec-11*E*-enoate.

**TABLE 2:** **Quantitative analyses of epidermal 9-HODE, 9-epoxyalcohol, and triol-3 in human epidermis**

Epidermal content (per g wet weight of epidermis)	9-HODE	9,10-*trans*-Epoxyalcohol*^a^*	Triol-3
nmol/g, *n* = 6 (range)	28.8 (20.9–37.1)	4.1 (1.7–5.4)	3.1 (2.1–3.8)
μg/g, *n* = 6 (range)	8.5 (6.1–10.9)	1.2 (0.5–1.7)	1.0 (0.7–1.2)

*^a^* The epoxyalcohol assayed is 9*R*,10*R-trans*-epoxy-13*R*-hydroxy-octadec-11*E*-enoate.

Unlike the epoxyalcohol and triols that are present almost entirely in esterified form, about half the HODEs measured in human epidermis are present as the free acid, non-esterified (supplemental Fig. S3). Therefore, the quantitative analysis of 9-HODE in Fig. 10*A* (the KOH-treated extractable lipids) represents free plus esterified HODEs; 9-HODE PFB ester eluted as the distinct peak at ∼10.5 min, about 0.2 min before the *d*4–9-HODE standard on this SP-HPLC system; other HODE isomers eluted in the *m*/*z* 295 (*d*0) channel, with 13-HODE at ∼8.5 min being prominent in all samples. Chiral HPLC analysis indicated the 9-HODE was predominantly 9*R* in configuration (supplemental Fig. S4). The presence of 9*R*-HODE along with 13*S*-HODE was reported in human psoriatic scales (38); 13*S*-HODE was identified as the more prominent product formed in homogenates of normal skin (39, 40).

The epoxyalcohol of human skin was almost exclusively esterified (Fig. 10*B*), with levels of the free products giving *d*0/*d*4 ratios similar to the *d*4 standard (supplemental Fig. S5). Fig 10*B* also illustrates the retention time of the later-eluting *cis*-epoxy isomer, 9*R*,10*S-cis*-epoxy313*R*-hydroxy-11*E*-octenoate. Enzymatic hydrolysis of this *cis-*epoxide would be expected to give rise to predominantly triol-1 as hydrolysis product, but it consistently appeared as a small peak relative to the *trans*-epoxide and apparently insufficient to account for the prominence of esterified triol-1 in the human epidermal extracts (*cf*. Fig. 8, *B* and *C*). Chiral HPLC analysis of the major *trans*-epoxyalcohol in three human samples gave values of 88, 97, and 98% of the 9*R*,10*R-trans*-epoxy-13*R*-hydroxy enantiomer (supplemental Fig. S6).

Assay of triol-3 in human epidermis confirmed its occurrence along with about half the amount of triol-1 (Fig. 10*C*). Similar to its epoxyalcohol precursor, the levels of free (non-esterified) triol-3 were below the limit of detection (supplemental Fig. S7). For comparison with the epidermal content of oxidized linoleate products, esterified LA, α-LNA, and AA concentrations were quantified as 9800 (range 8500–12,800) μg/g, 1523 (1200–1800) μg/g, and 1423 (1000–2050) μg/g, respectively. Remarkably, the oxidized products are present in human epidermis in ∼0.01% of the abundance of intact linoleate (for epoxyalcohol and triol) and 0.06% relative abundance (for 9-HODE and 13-HODE). Nonetheless, the oxidized products are chiral, indicating their enzymatic origin and genetic evidence confirms the importance of their biosynthesis in the mammalian epidermal barrier.

##### Examining an Alternative Route to Forming Protein-bound Ceramide

**SCHEME 2.**
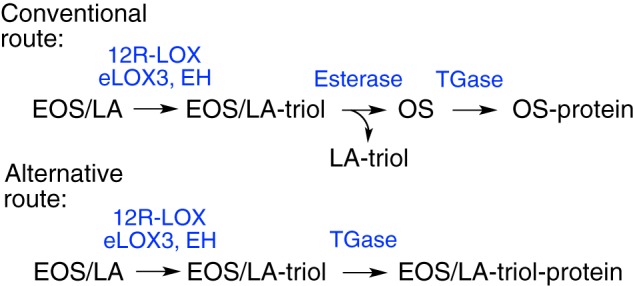
**Conventional concept and alternative mode of ceramide coupling to protein.** Routes to protein-bound ceramide: conventional coupling via the ω-hydroxyl of ceramide OS or an alternative via a hydroxyl of LA-triol. *EH,* epoxide hydrolase; *TGase*, transglutaminase.

As noted in the Introduction, the ultimate structural consequence of the oxidations in the LOX pathway of the epidermal barrier is formation of protein-bound ceramide and the CLE. The conventional view, as we proposed, is that oxidation of the linoleate esterified in ceramide-EOS facilitates hydrolysis of the ester bond, releasing ceramide-OS for coupling to protein via its free ω-hydroxyl (Scheme 2, conventional route) (8). That the OS ceramide is directly coupled to protein is supported by mass spectrometric evidence of ceramide-peptide conjugates isolated from epidermal proteins and by the coupling of OS surrogates to protein via transglutaminase (41, 42). Nonetheless, these experiments do not fully account for the total protein-bound ceramide (41, 43), and the oxidation of linoleate to triol has the potential for protein binding via a hydroxyl of the linoleate triol, obviating the need for the (so far unidentified) esterase cleavage of the oxidized linoleate (Scheme 2, alternative route).

To test for protein-bound triols, pig epidermis was extracted several times with CHCl_3_/MeOH, including soaking overnight in the solvents. The washed protein pellet was then treated with KOH overnight to hydrolyze ester bonds and to release protein-bound fatty acids. An aliquot of *d*4-triols was then added, and the samples were taken through the usual extraction and derivatization procedures for LC-MS analysis of the PFB-DMP triols. A sample of the solvent-extracted lipids (with added d4-triol mixture) was analyzed in parallel. In contrast to the strikingly prominent peak of triol-3 recovered from the soluble esters, the pattern of triols released from the protein pellet was largely a nonspecific mixture of all eight triols with a slight preponderance of triol-3 (supplemental Fig. S8). Furthermore, there was greater than 10-fold excess of triol-3 esterified in the CHCl_3_/MeOH-soluble lipids compared with the amounts recovered from the protein pellet. We conclude that the triol-3 could likely arise from lipid retained in the protein pellet. The rest of the mixture is a non-selective hydrolysis of *RRR* and *RRS* epoxyalcohols (Fig. 4, *top*), the latter not an enzymatic product, and therefore we deduce that the mixture reflects a low level of non-enzymatic oxidation and is not a significant participant in the binding of ceramides to protein.

## Discussion

### 

#### 

##### Detection of Esterified Triols and Their Significance

**FIGURE 11.**
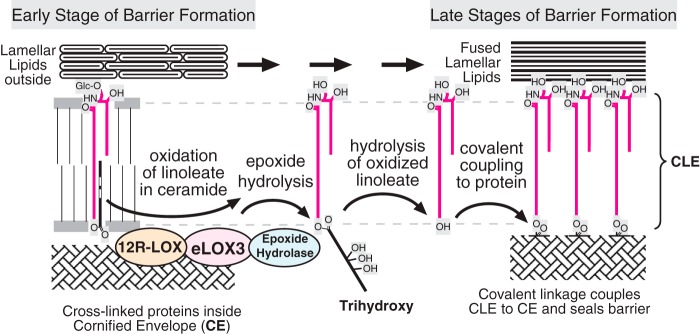
**Synthesis of linoleate triols in ceramide esters and their role in formation of the CLE.** Early in barrier construction lamellar granules fuse with the corneocyte plasma membrane leaving Glc-EOS spanning the membrane (*left side*) (56). Ultimately, the phospholipids are cleared, being replaced by ceramides, although free OS (now de-glucosylated) is covalently coupled to the polymerized protein of the corneocyte envelope, thus forming the CLE (*right side*). CLE formation is facilitated by oxidation of the linoleate moiety of EOS (*black chain*) by 12*R*-LOX and eLOX3; putative epoxide hydrolase-catalyzed epoxide hydrolysis then yields esterified linoleate-triol, the polar structure of which disrupts the lipid structures and facilitates de-esterification of the EOS-type ceramides, providing the free OS for coupling to protein and thus creating the CLE.

Our findings establish the existence of trace levels of esterified linoleate triols in human and pig epidermis, which is consistent with their obligatory role in forming the intact skin water barrier. Despite the tiny percentage (≤0.1%) compared with the esterified linoleate itself, the abundance of specific isomers, and particularly the chiral analyses, leaves no doubt that these linoleate triols derive from specific enzymatic oxidation. Their pure 9*R* chirality is characteristic of 12*R*-LOX oxidation of linoleate, and the pure 13*R* chirality matches the product of eLOX3 transformation of 9*R*-HPODE to the epoxyalcohol 9*R*,10*R-trans*-epoxy-13*R*-hydroxy-octadec-11*E*-enoate (8). We should emphasize that only trace levels of these specific triols are to be expected according to our hypothesis of their role in differentiation of the skin barrier. By the disrupting effect on the lipid environment, it is proposed that the polar trihydroxy-linoleates facilitate the actions of an esterase that cleaves the triol (and probably facilitating the hydrolysis of other EOS species, too) to produce free OS for covalent coupling to protein and construction of the CLE (Fig. 11). This is another example of the well accepted line of evidence that oxidation of polyunsaturated esters leads to their preferential cleavage from lipid membranes (44–47). Therefore, the oxidized linoleates are intermediates in ongoing transformations that lead to their removal from the membrane, and accordingly, the detection of trace levels is consistent with their obligatory role in barrier function.

##### Specific Triol Structures and Their Mechanism of Formation

It is well established that non-enzymatic hydrolysis of the eLOX3-derived allylic epoxyalcohol produces a mixture of four triols formed by attack of water at the 10- and 12-carbons, the four varying in relative abundance from ∼10 to 60% (Fig. 3*A*) (*cf*. Refs. 35, 48, 49). By contrast, pig epidermis contains only one prominent triol esterified in the lipids, accounting for around 90% of the total. The same isomer is also the most abundant in human epidermis. The major esterified triol we identify is the expected product of a specific S_N_2 hydrolysis that reverses the configuration at C-10 of its 9*R*,10*R*,13*R* epoxyalcohol precursor, giving the 9*R*,10*S*,13*R*-trihydroxy-octadec-11*E*-enoate. In human epidermis this is accompanied by lesser amounts of the 10*R* isomer. A potential explanation for the occurrence of the second triol (9*R*,10*R*,13*R*) in human epidermis would be the presence of a 9*R*,10*S-cis*-epoxy-13*R*-hydroxy precursor in the lipids, the *S*_N_2 hydrolysis of which would form the *RRR* triol. However, the peak of this *cis*-epoxide detectable in our LC-MS analyses is minor (Fig. 10*B*) and appears insufficient to account for the relative prominence of the *RRR* triol. Our studies with metabolism of 9*R*-HPODE by recombinant human eLOX3 support formation of only the 9*R*,10*R-trans*-epoxy-13*R*-hydroxy epoxyalcohol (8). To the best of our knowledge, there is no precedent for non-enzymatic hydrolysis in which shielding might eliminate the attack of water at C-12, and therefore involvement of epoxide hydrolase(s) is the most straightforward explanation for occurrence of the specific linoleate triols in the esterified lipids.

##### Possible Involvement of Epoxide Hydrolase

Hydrolysis catalyzed by an epoxide hydrolase would be specific, with pure *S*_N_2 attack on the allylic 10-carbon, thus producing the major triol we find esterified in pig and human epidermis. So far, no epoxide hydrolase has been tested with substrates directly relevant to our study. In terms of known catalytic activities, mammalian soluble epoxide hydrolase (sEH, EPHX2) is a potential candidate as it efficiently hydrolyzes *trans-*epoxides, and the *trans-*epoxides of arachidonic acid-derived hepoxilins are good substrates (50, 51). In the same gene family is also the microsomal epoxide hydrolase (mEH, EPHX1), which has specificity for *cis*-epoxides (52) and therefore appears an unlikely candidate. For epoxide hydrolase 3 (EPHX3, ABHD9), so far high catalytic activity is demonstrated for non-allylic *cis*-epoxy-fatty acids, epoxy-stearate, and epoxy-eicosatrienoic acids (53); the suitable catalytic activity of EPHX3 on the allylic *trans*-epoxyalcohols remains an open issue. Currently, there is no information on the substrate specificities of the other two putative epoxide hydrolases, epoxide hydrolase 4 (EPHX4 or ABHD7) and MEST/Peg1 (paternally expressed gene-1) (52, 54), nor on several members of the more distantly related mammalian α-β-hydrolases implicated as acyltransferases or hydratases (54).

Expression in skin or epidermis, as far as established, is suitable for sEH (55) and EPHX3 (53) and unknown/not investigated for most other candidates. EPHX3 was detected as a protein highly expressed in human outer epidermis (57) and identified in an ichthyosis gene cluster by comparative mRNA coexpression (58). Despite such indications, inactivating mutation of the murine and human EPHX3 gene give no reported skin phenotypes (59) and similarly for knockouts of mEH (60), sEH (61), and MEST/peg1 (62). So based on the information currently available, it appears that the current murine gene knock outs do not target the appropriate epoxide hydrolase, or alternatively there may be redundancy in the epoxide hydrolase function in the epidermis.

##### Product Polarity as the Key Feature of Linoleate Oxidation

We surmise the primary role of the linoleate triols in barrier formation is to induce disruption of the membrane lipids, in turn facilitating the ester hydrolysis that provides OS ceramide for construction of the CLE. The actions of 12*R*-LOX convert the relatively non-polar linoleate to the 9-hydroperoxide with a significant increase in polarity of the oxidized linoleate ester. Why then does eLOX3 gene knock-out (*Aloxe3*^−/−^) result in a significant skin phenotype? The further action of eLOX3 transforms the 9-hydroperoxide to an epoxyalcohol, which does not itself induce a major increase in polarity, yet eLOX3 gene deletion is associated with a strong phenotype. We conclude it is the further transformation of epoxyalcohol to trihydroxy-linoleate that the eLOX3 reaction permits that explains the significant phenotype when *Aloxe3* is deleted. The *Aloxe3*^−/−^ phenotype is slightly less severe than the 12*R*-LOX knock out (7), and one might anticipate that deletion of the epoxide hydrolase(s) responsible for linoleate triol synthesis to have a similar phenotype.

##### History of Linoleate Oxidations and the Epidermal Barrier

In the mid-1980s Nugteren *et al.* (63) reported the metabolism of [1-^14^C]linoleic acid applied topically to the back skin of EFA-deficient rats. Identified among the radiolabeled products were what they termed “polyoxyacyl” ceramides, the structures represented as a mixture of 9,10,11-, 9,10,13-, 9,12,13-, and 11,12,13-trihydroxy-linoleates esterified to EOS ceramide. The authors concluded that formation of these derivatives is important for the integrity of the epidermal water barrier, and in a subsequent review they sum up as follows: “Currently, however, we believe that it is the polyoxyacylceramide derivative of AC (acylceramide or EOS), recently discovered by ourselves, which is key to the correct formation or maintenance of the permeability barrier in the compactum region. How it achieves this remains a mystery and is currently under investigation” (64). As it happened, no new insights were forthcoming, and furthermore, others discounted the findings of oxidized linoleate in the epidermal ceramides as non-enzymatic artifacts (65). Nothing more has appeared on the subject until our characterizations of specific oxidation of linoleate in the epidermal EOS (8) and extended now to identify the very specific LOX-derived linoleate triols. Clearly, our findings support the early work and conclusions of Nugteren *et al.* (63) and provide the missing conceptual model that links the EFA metabolism to construction of the epidermal water barrier.

## Author Contributions

T. C., C. P. T., V. B. O., and A. R. B. designed the study. T. C. performed the experiments shown in Figs. 5–10; C. P. T. performed, analyzed, and wrote the text of experiments associated with Fig. 2; A. R. B. and M. W. C. contributed to development and validation of the quantitative LC-MS analyses and performed the experiments in supplemental Figs. 3–8; W. E. B. prepared the linoleate 9-hydroperoxides, epoxyalcohols, and triol standards; T. C. and A. R. B. wrote the manuscript and prepared the figures; all authors read and edited the manuscript.

## Supplementary Material

Supplemental Data

## Footnotes

4 The abbreviations used are:

LOX: lipoxygenase
APCI: atmospheric pressure chemical ionization
CLE: corneocyte lipid envelope
DCM: dichloromethane
EFA: essential fatty acid
EPHX: epoxide hydrolase
eLOX3: epidermal lipoxygenase-3
EOS (or EOH, EOP): esterified omega-hydroxyacylsphingosine (or hydroxysphingosine, phytosphingosine)
Glc-EOS: acylglucosylceramide
HAc: glacial acetic acid
H(P)ODE: hydro(pero)xy-octadecadienoic acid
IPA: isopropyl alcohol
SIM: selection ion monitoring
OS: omega-hydroxyacylsphingosine
SIM: selection ion monitoring
SP-HPLC: straight-phase HPLC
DMP: 2,2-dimethoxypropane
PFBBr: pentafluorobenzyl bromide
AA: arachidonic acid
LA: linoleic acid
LNA: α-linolenic acid
PFB: pentafluorobenzyl
mAU: milli-absorbance unit.
