# Supplementary material for: The Precise Structures and Stereochemistry of Trihydroxy-linoleates Esterified in Human and Porcine Epidermis and Their Significance in Skin Barrier Function: IMPLICATION OF AN EPOXIDE HYDROLASE IN THE TRANSFORMATIONS OF LINOLEATE
Source: J Biol Chem. 2016 May 5;291(28):14540–54. doi: 10.1074/jbc.M115.711267 (PMC4938176; doi:10.1074/jbc.M115.711267)
Supplement: Supplemental Data [file supp_291_28_14540__index.html]

The precise structures and stereochemistry of trihydroxy-linoleates esterified in human and porcine epidermis and their significance in skin barrier function: IMPLICATION OF AN EPOXIDE HYDROLASE IN THE TRANSFORMATIONS OF LINOLEATE — The precise structures and stereochemistry of trihydroxy-linoleates esterified in human and porcine epidermis and their significance in skin barrier function: IMPLICATION OF AN EPOXIDE HYDROLASE IN THE TRANSFORMATIONS OF LINOLEATE — The Precise Structures and Stereochemistry of Trihydroxy-linoleates Esterified in Human and Porcine Epidermis and Their Significance in Skin Barrier Function — Linoleate Triols in Skin Barrier Function — Supplemental Data 

# The Precise Structures and Stereochemistry of Trihydroxy-linoleates Esterified in Human and Porcine Epidermis and Their Significance in Skin Barrier Function

## Supplemental Data

- Supplement (.pdf, 277 KB) - Supplementary figures S1 - S8
