# Supplementary material for: The Precise Structures and Stereochemistry of Trihydroxy-linoleates Esterified in Human and Porcine Epidermis and Their Significance in Skin Barrier Function: IMPLICATION OF AN EPOXIDE HYDROLASE IN THE TRANSFORMATIONS OF LINOLEATE
Source: J Biol Chem. 2016 May 5;291(28):14540–54. doi: 10.1074/jbc.M115.711267 (PMC4938176; doi:10.1074/jbc.M115.711267)

SUPPLEMENTAL DATA

The Precise Structures and Stereochemistry of Trihydroxy-Linoleates Esterified in Human and Porcine Epidermis and Their Significance in Skin Barrier Function: IMPLICATION OF AN EPOXIDE HYDROLASE IN THE TRANSFORMATIONS OF LINOLEATE

Takahito Chiba, Christopher P. Thomas M. Wade Calcutt, William E. Boeglin,
Valerie B. O'Donnell, and Alan R. Brash

Fig. S1. Analytical procedures for the identification of free and esterified products in epidermis

Fig. S2. Analytical procedures for the quantitation of free and esterified 9-HODE, epoxyalcohol (EpOH) and triols in epidermis.

Fig. S3 LC-MS analysis of the free (non-esterified) 9-HODE in human epidermis

Fig. S4 Chiral analysis of 9-HODE in human epidermis

Fig. S5 LC-MS analysis of the free (non-esterified) linoleate epoxyalcohol in human epidermis

Fig. S6 Chiral column LC-MS analysis of the stereochemistry of the major epoxyalcohol esterified in human epidermis

Fig. S7 LC-MS analysis of the free (non-esterified) linoleate triol in human epidermis

Fig. S8 The profile of protein-bound triols in pig epidermis

Figure S1. **Analysis of free and esterified products in epidermis**
Purification scheme for analysis of triols in human and pig epidermis. After extraction from epidermis using $CHCl_3$/MeOH and open-bed silica fractionation, separate aliquots were analyzed for free triols (left-hand arm) and esterified triols (middle and right-hand arms). Esterified triols were released by alkaline hydrolysis overnight. After Bligh & Dyer partitioning to remove neutral and non-polar lipids in the $CHCl_3$ phase, samples were either extracted on an Oasis HLB cartridge at pH 6 (to avoid hydrolysis of epoxyalcohols to triols), or after standing at room temperature at pH 3 for 30 min to allow acid hydrolysis of epoxyalcohols. The recovered triols were converted to the PFB DMP derivative and analyzed by HPLC, GC-MS and LC-MS.

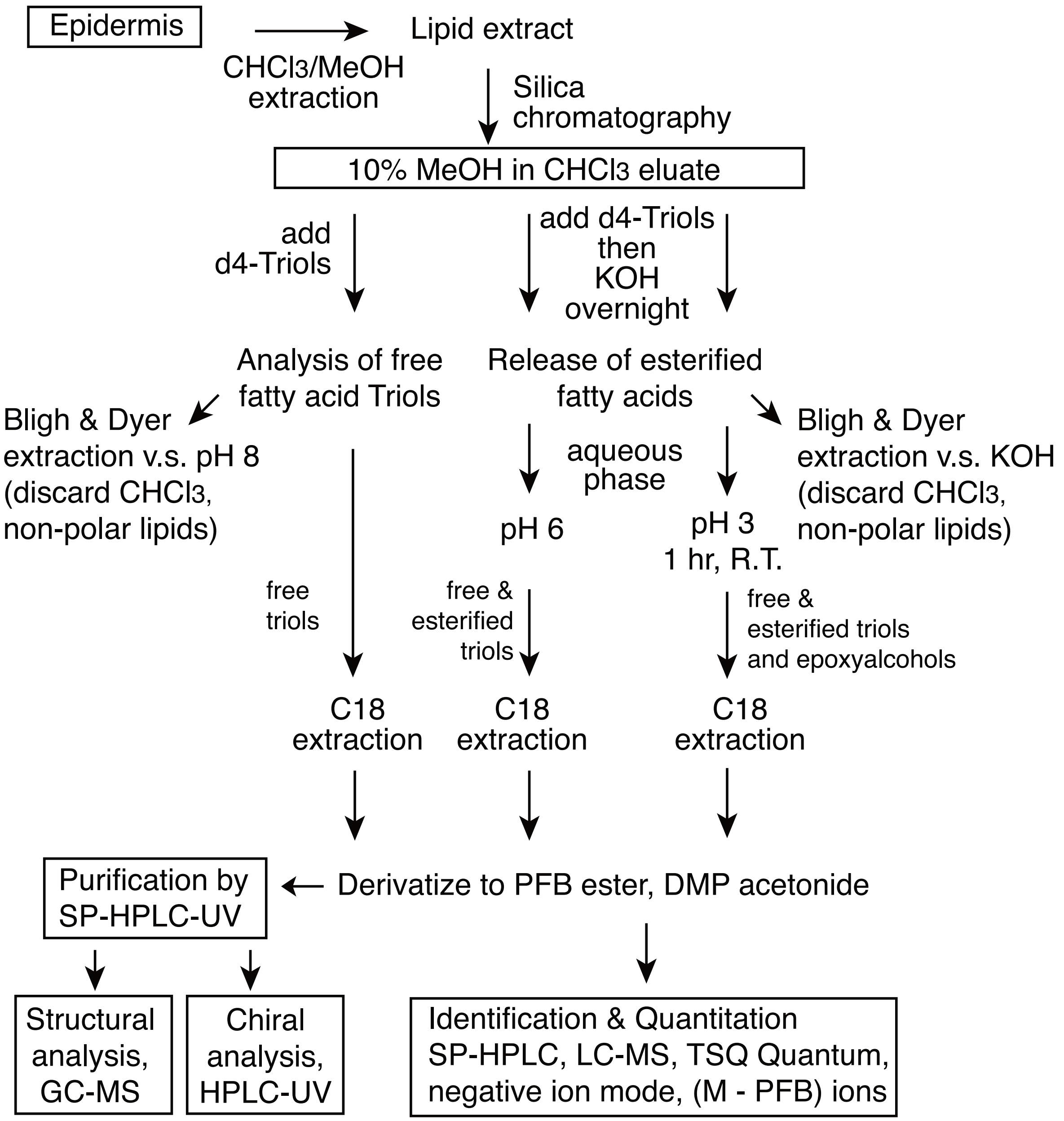

Figure S2. Quantitation of free and esterified 9-HODE, epoxyalcohol (EpOH) and triols in epidermis.
After the initial extraction and open-bed silica column, free fatty acid products are taken through the left-hand arm of the scheme; Bligh and Dyer partition using 0.1 M K_2HPO_4 as the aqueous phase allows removal of neutral and non-polar lipids in the $CHCl_3$ while retaining the ionized fatty acids in the aqueous layer; the free acids in the mildly alkaline solution are subsequently retained on an Oasis HLB cartridge, eluted with EtOAc and derivatized for LC-MS analyses. The right-hand arm follows a similar protocol after esterified products are released by KOH treatment overnight.

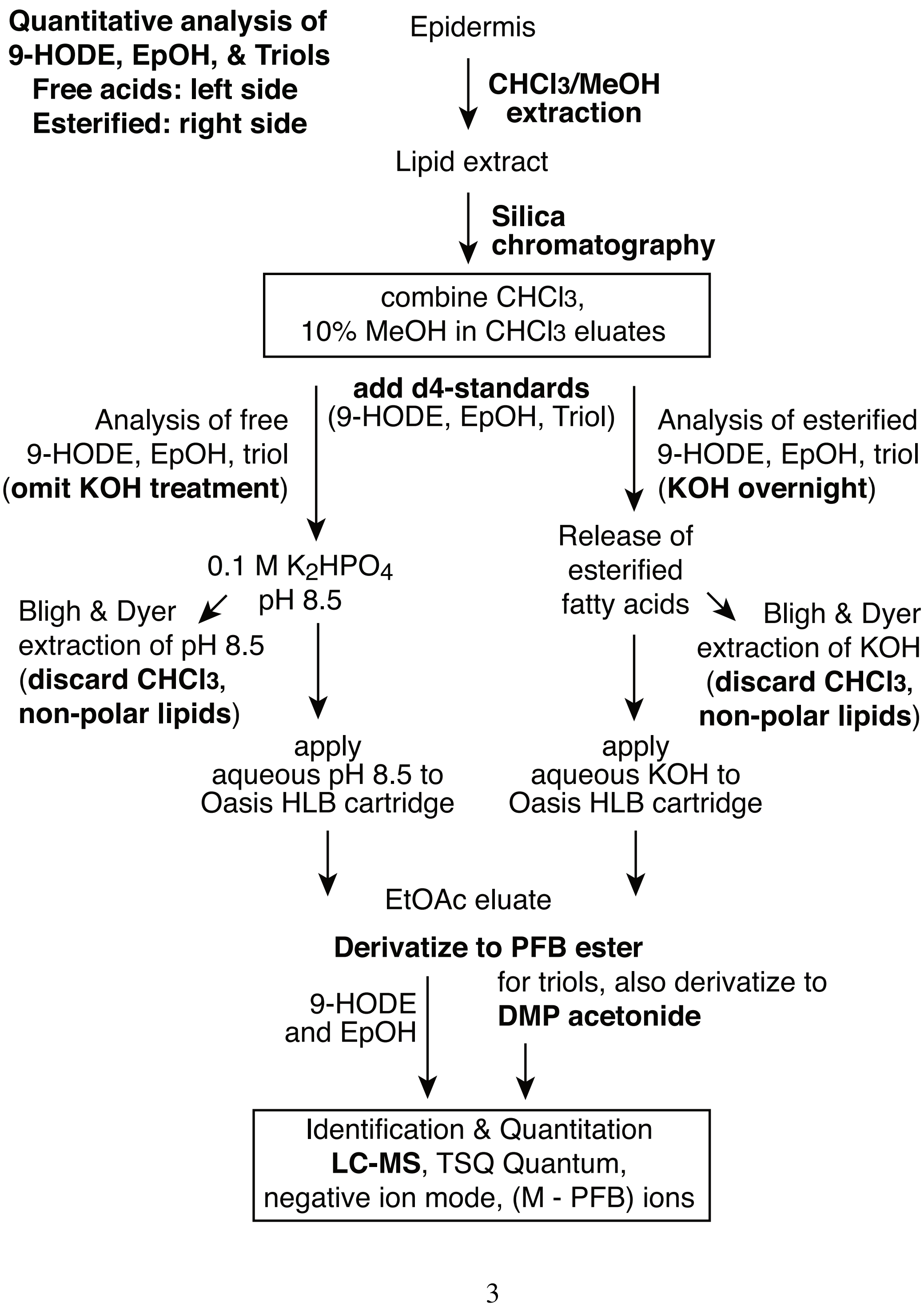

Fig S3: LC-MS analysis of the free (non-esterified) 9-HODE in human epidermis
The analyses used a Thomson Scientific Advantage 5μ silica column (25 x 0.46 cm), with a solvent of solvent hexane/IPA (100:0.5 v/v), flow rate 1 ml/min, and LC-MS detection of the PFB ester (M – PFB ion) at m/z 295 (do) and 299 (d4). The early peak at ~6.6 min in the m/z 295 channel (d0) is 13-HODE.
The chromatogram is representative of the epidermis from three different subjects.

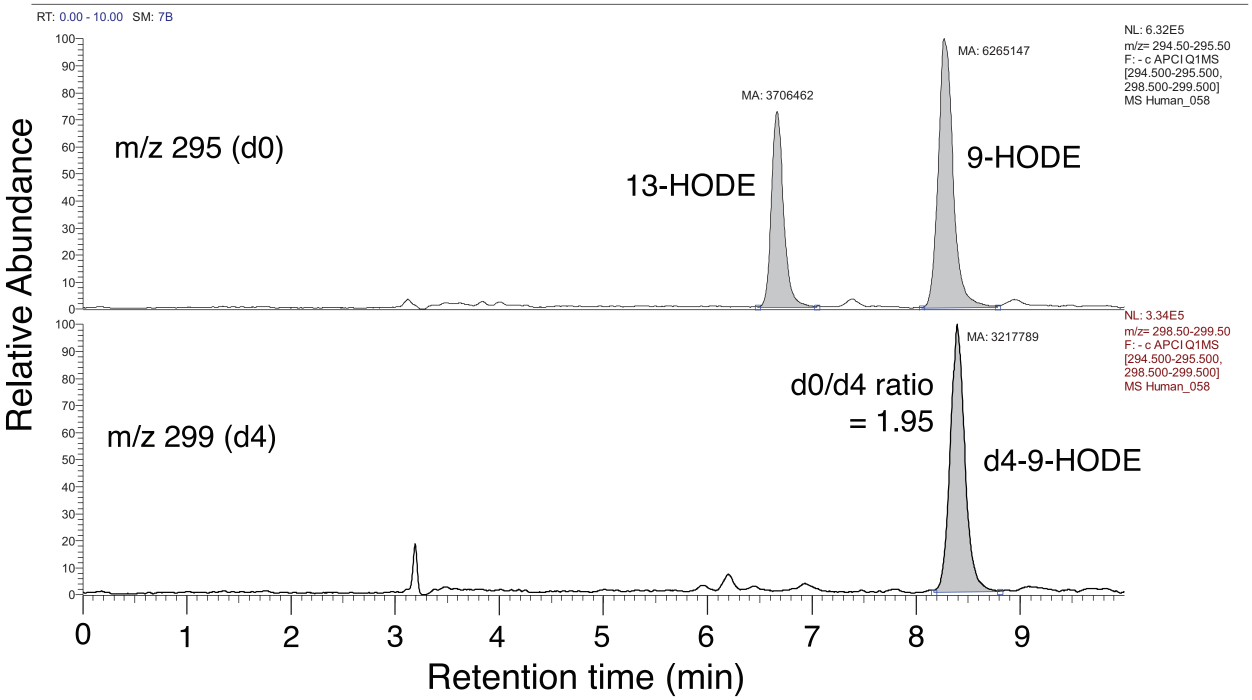

Fig. S4: Chiral HPLC analysis of 9-HODE from human epidermis
The analyses used a Chiralcel OD-H column (25 x 0.46 cm), a solvent of hexane/IPA (100:5 v/v) and a flow rate of 1 ml/min with UV-detection of the conjugated diene chromophore at 235 nm. Epidermis from three different individuals were analyzed: the percentage of 9*R* enantiomer was 79.8%, 85.5% (shown above), and 85.7%.
The analyses were conducted on the total 9-HODE (free plus esterified).

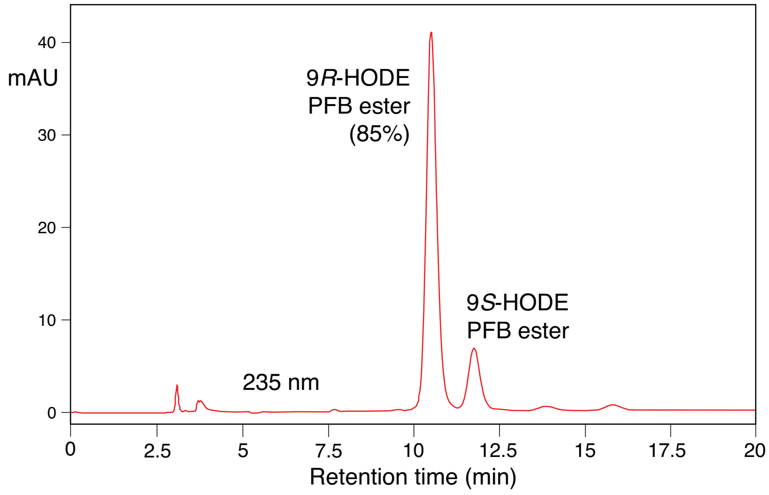

Fig. S5: LC-MS analysis of the free (non-esterified) epoxyalcohol in human epidermis

The ratio of d0/d4 epoxyalcohol in this analysis is 0.0025, corresponding to only ~2% of the esterified epoxyalcohol.
(By comparison, the d0/d4 ratio measured for <u>esterified</u> epoxyalcohol is approximately 0.5, Fig. 10B).

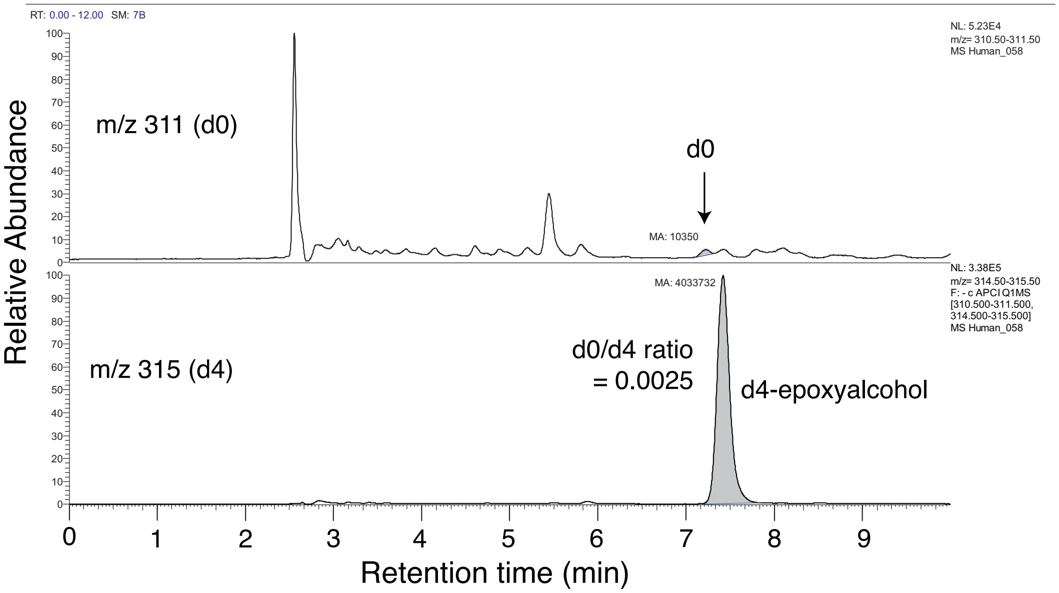

Fig S6: Chiral column LC-MS analysis of the chirality of the major epoxyalcohol esterified in human epidermis as 9*R*,10*R*-trans-epoxy-13*R*-hydroxy-octadec-11*E*-enoate.
The analysis used a Chiralcel OJ column (25 x 0.46 cm), a solvent of hexane/IPA (100:10 v/v), a flow rate 1 ml/min, with LC-MS detection of PFB ester monitoring the [M – PFB]- ion at m/z 311.
Epidermis from three different individuals were analyzed: the percentage of *RRR* enantiomer was 88%, 97% (shown above), and 98%.

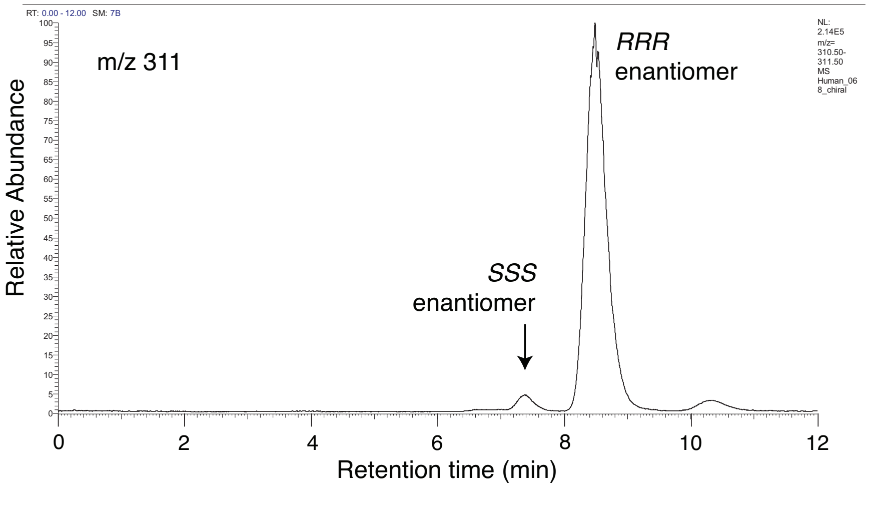

Fig. S7 LC-MS analysis of the free (non-esterified) linoleate triols in human epidermis
The samples were analyzed for free (non-esterified) linoleate triols using the method summarized in Fig 2B of the main text. Detection of the PFB ester, DMP derivative for unlabeled triols (m/z 369) and the d4-triol-3 and d4-triol-4 detected at m/z 373.
(By comparison, the d0/d4 ratio measured for esterified triol-3 is approximately 220 parts per thousand, Fig. 10C).
The peak at ~ 7.7 min below in the d0 channel is a contaminant of pinellic acid (triol-2) used to check the derivatization.

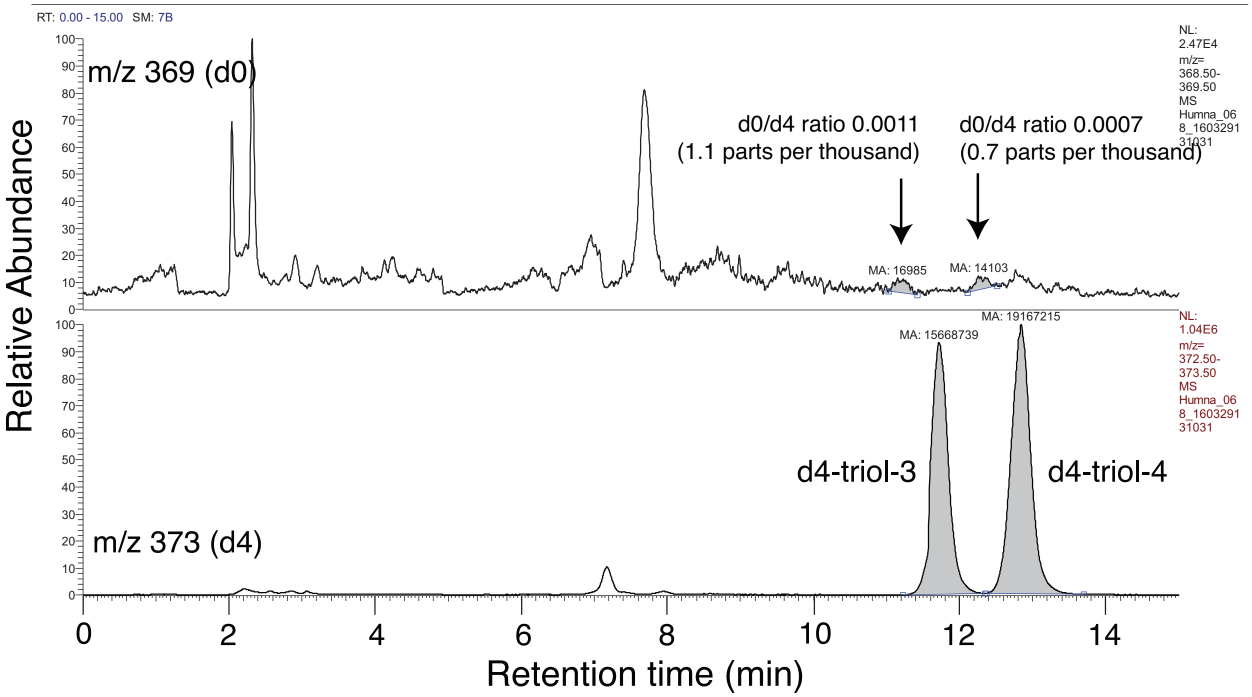

Fig. S8 (A, top) The profile of protein-bound triols in pig epidermis compared with $CHCl_3$/MeOH soluble esterified triols (B, below).

Normal-phase LC-MS analysis of the PFB ester DMP acetonide derivative using a Thomson Advantage 5 µ silica column with a solvent of hexane/IPA (100:1, v/v), a flow rate of 1 ml/min with monitoring of the $[M – PFB]^-$ ion at m/z 369.

The numbers correspond to the linoleate triols shown in Fig. 3 of the main text. The intensity of the major esterified triol-3 (lower panel) was over 10-fold higher than the corresponding protein-bound triol.

We conclude that non-specific oxidation accounts for these comparatively low levels of protein bound triols and that coupling of EOS-triol to protein (the a hydroxyl on the triol moiety) is of minor consequence compared to protein binding via the omega-OH of ceramide OS.

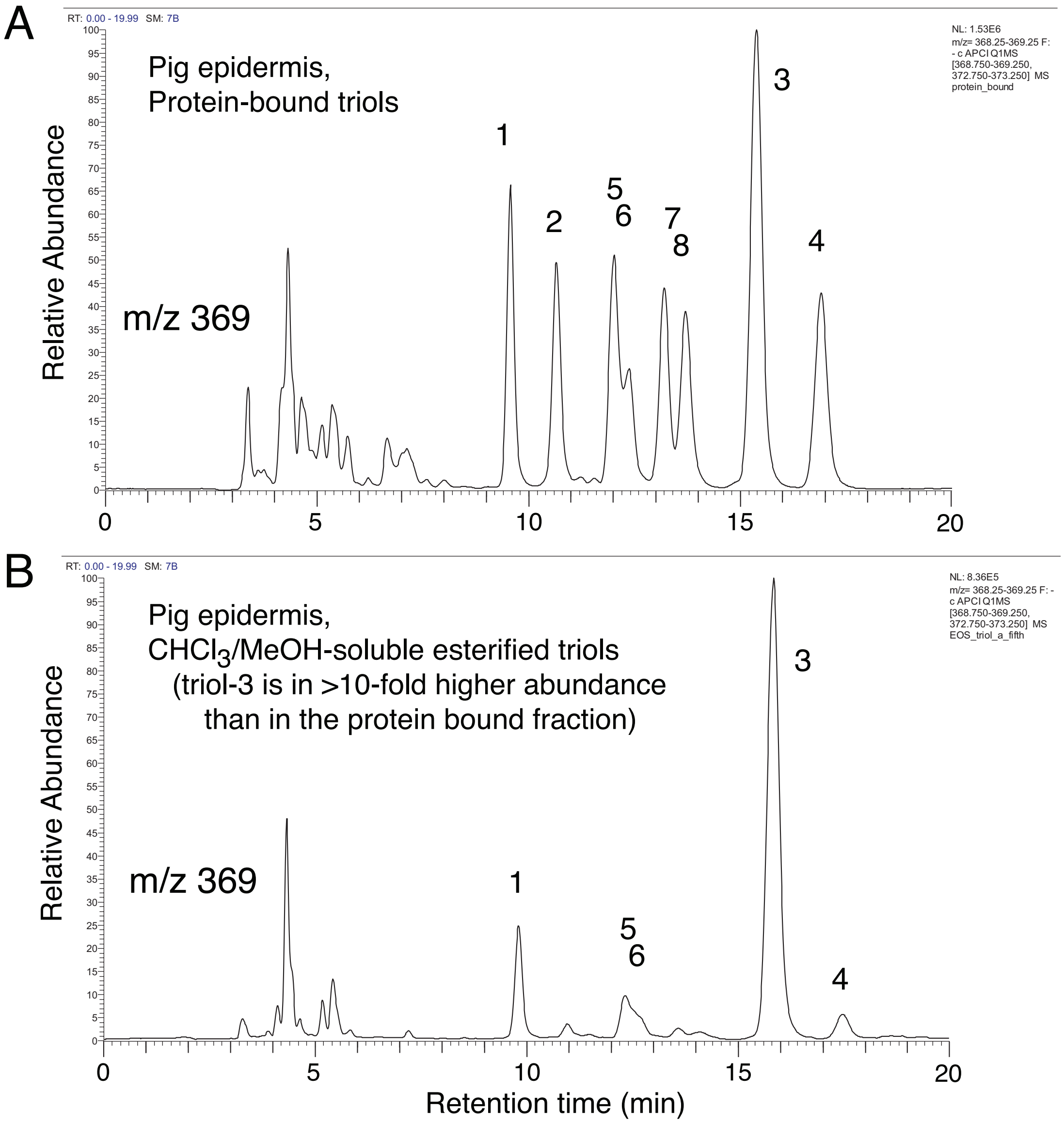

Supplement: Supplemental Data [file 10.1074_M115.711267_jbc.M115.711267-1.pdf]
